# RBCK1 promotes hepatocellular carcinoma metastasis and growth by stabilizing RNF31

**DOI:** 10.1038/s41420-022-01126-x

**Published:** 2022-07-22

**Authors:** Xijun Chen, Qing Ye, Wenxiu Zhao, Xiaoqin Chi, Chengrong Xie, Xiaomin Wang

**Affiliations:** 1 The Third Clinical Medical College, Fujian Medical University, Fuzhou, China; 2 Department of Plastic Surgery, The First Affiliated Hospital of Fujian Medical University, Fuzhou, Fujian P. R. China; 3 Xiamen Translational Medical Key Laboratory of Digestive System Tumor, Fujian Provincial Key Laboratory of Chronic Liver Disease and Hepatocellular Carcinoma, Zhongshan Hospital of Xiamen University, School of Medicine, Xiamen University, Xiamen, China

**Keywords:** Cancer, Biochemistry

## Abstract

RNF31 (HOIP), RBCK1 (HOIL-1L), and SHARPIN are subunits of the linear ubiquitin chain assembly complex. Their function and specific molecular mechanisms in hepatocellular carcinoma (HCC) have not been reported previously. Here, we investigated the role of RNF31 and RBCK1 in HCC. We showed that RNF31 and RBCK1 were overexpressed in HCC and that upregulation of RNF31 and RBCK1 indicated poor clinical outcomes in patients with HCC. RNF31 overexpression was significantly associated with more satellite foci and vascular invasion in patients with HCC. Additionally, RBCK1 expression correlated positively with RNF31 expression in HCC tissues. Functionally, RBCK1 and RNF31 promote the metastasis and growth of HCC cells. Moreover, the RNF31 inhibitor gliotoxin inhibited the malignant behavior of HCC cells. Mechanistically, RBCK1 interacted with RNF31 and repressed its ubiquitination and proteasomal degradation. In summary, the present study revealed an oncogenic role and regulatory relationship between RBCK1 and RNF31 in facilitating proliferation and metastasis in HCC, suggesting that they are potential prognostic markers and therapeutic targets for HCC.

## Introduction

Hepatocellular carcinoma (HCC) exhibits highly malignant biological behavior and causes a large number of tumor-related deaths [1]. Over the past several decades, surgical treatments, percutaneous radiofrequency ablation, hepatic artery infusion chemotherapy, and immunotherapy have improved the short- and long-term outcomes in HCC patients. Nevertheless, the prognosis of patients with HCC remains poor due to metastasis and recurrence [2]. Therefore, there is an urgent need for active research into HCC tumorigenesis and progression mechanisms to provide clinical therapeutic strategies.

The linear ubiquitin chain assembly complex (LUBAC) consists of ring finger protein 31 (RNF31, also known as HOIP), RANBP2-type, C3HC4-type zinc finger-containing 1 (RBCK1, also known as HOIL-1L), and SHANK-associated RH domain interactor (SHARPIN), which are the only E3 ubiquitin ligases currently known to participate in the formation of linear ubiquitin linkages [3]. Previous studies have shown that the LUBAC contributes to the regulation of numerous physiological and pathological processes, such as inflammation [4], autoimmunity [5–7], and the cell cycle [8], which are relevant to cancer progression. The LUBAC plays important roles in cancer development and progression, including those of prostate [9], renal [10], and breast cancers [11]. A previous study reported that SHARPIN facilitates HCC development [12]. However, to our knowledge, the function and related molecular mechanisms of RBCK1 and RNF31 in HCC have not been investigated.

RNF31 is a zinc finger protein that is a member of the Paul subfamily of RING-in-between-RING (RBR) ubiquitin ligase E3. Only the RBR structural domain of RNF31 regulates linear ubiquitination modifications, which is distinct from that of RBCK1 [13, 14]. RNF31 participates in a variety of physiological functions and pathophysiological activities, including evasion of apoptosis [15], T cell function [16], proliferation ability, protein stability [17], and metastasis [18]. Furthermore, RNF31 plays an important role in various solid tumors, such as prostate [19], gastric [16], colon [20], and breast cancers [21], in which its levels are elevated, in association with a dismal prognosis, by multiple mechanisms. For instance, in breast cancer, RNF31 interacts with ERα, supporting its mono-ubiquitination and enhancing Erα stability, thereby promoting the growth of breast cancer [17]. RNF31 also interacts with P53 and promotes its ubiquitinated degradation [11]. To date, little is known regarding the role and mechanism of RNF31 in HCC, but understanding the role of RNF31 in HCC may be informative for both the diagnosis and treatment of this cancer.

RBCK1 is an RBR E3 ligase [22]. RBCK1 has been reported to be overexpressed in lung adenocarcinoma [23], breast cancer [24], colorectal cancer [25], and renal cell carcinoma [26, 27], where high RBCK1 expression correlates with an unfavorable prognosis. RBCK1 promotes tumor progression through several mechanisms. As an E3 ligase, RBCK1 promotes P53 degradation in renal cancer by catalyzing its ubiquitination [26]. Moreover, RBCK1 facilitates PTEN degeneration via ubiquitination in ovarian cancer [28]. Recently, RBCK1 has been identified as a unique E3 that causes ester-linked ubiquitination in Ser/Thr residues [29]. Nevertheless, the role of RBCK1 in HCC development has not been investigated to date.

Here, we investigated the roles of RNF31 and RBCK1 in HCC and investigated the mechanisms by which they exert their functions in this context.

## Results

### RNF31 is overexpressed in human HCC tissues and its expression correlates with patient prognosis

**Fig. 1:**
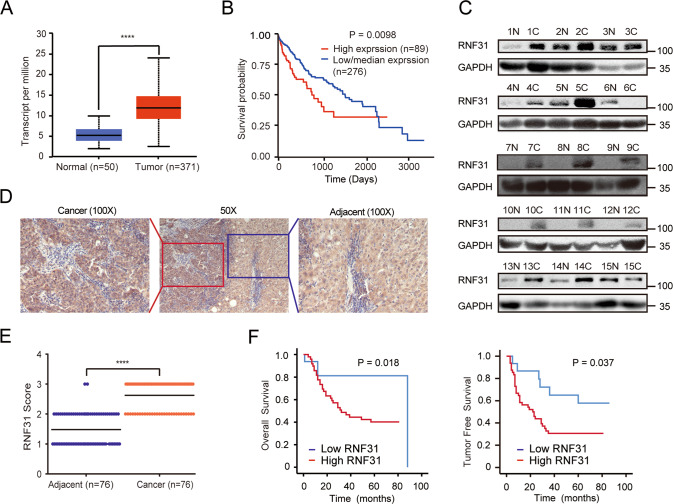
RNF31 is overexpressed in human HC tissues and its expression correlates with patient prognosis. **A** *RNF31* mRNA expression in human HCC tissues and normal liver tissues from TCGA database. **B** Kaplan–Meier analysis of overall survival curves for the RNF31 low and high expression human HCC cases in the TCGA. **C** RNF31 protein expression in pairs of HCC and corresponding noncancerous liver tissues from 15 patients were analyzed by western blot. **D** Typical IHC picture of RNF31 in paired HCC and corresponding noncancerous liver tissues. **E** IHC assay was performed to detect RNF31 expression levels in HCC tissues and adjacent noncancerous liver tissues. Immunohistochemical score were analyzed using Wilcoxon’s test (*n* = 76, ****p < 0.001). **F** Overall survival and tumor-free survival of HCC patients were analyzed by Kaplan–Meier survival analysis (log-rank test). The patients were divided into two groups based on the immunohistochemical score of RNF31 in HCC.

By analyzing TCGA online database (http://ualcan.path.uab.edu/analysis.html), we found that the mRNA expression of RNF31 was higher in human HCC tissues than in normal liver tissues (Fig. 1A). Additionally, the upregulation of RNF31 increased the risk of poor prognosis (Fig. 1B). To validate this finding, we detected RNF31 protein expression in pairs of HCC and corresponding noncancerous liver tissues from 15 patients using western blotting and observed that 12/15 HCC tissues exhibited RNF31 elevation as compared to the adjacent noncancerous liver tissues (Fig. 1C).

An IHC assay was performed to detect RNF31 protein expression levels in HCC tissues and adjacent noncancerous liver tissues in 76 HCC patients. RNF31 was mainly expressed in the cytoplasm. In 85.5% of patients (65/76), RNF31 protein levels were higher in HCC tissues than in the adjacent noncancerous liver tissues (Fig. 1D, E). Moreover, Kaplan–Meier analysis demonstrated that RNF31 protein overexpression was negatively correlated with overall survival and tumor-free survival (Fig. 1F). Thus, RNF31 may be a significant prognostic factor for patients with HCC.

To further evaluate the clinical significance of RNF31, the potential correlation between RNF31 protein expression and clinicopathological parameters of patients with HCC was analyzed. As shown in Table 1, the high-RNF31 group exhibited more satellite foci (*p* = 0.003) and vascular invasion (*p* = 0.038). However, age, sex, tumor size, differentiation, alpha-fetoprotein, HBV DNA, and liver cirrhosis were not significantly correlated with RNF31 expression.

**Table 1:** Correlation between RNF31 score in HCC and clinicopathological factors.

Clinicopathological factors	RNF31 expression		*p* value
	Low	High	
Age (years)			
<60	6	16	0.7663
≥60	10	33	
Sex			
Male	11	43	0.1208
Female	5	6	
Tumor size (cm)			
≤5	9	36	0.2229
>5	7	13	
Differentiation level			
Low, medium	15	40	0.4290
High	1	9	
Satellite foci			
Without	1	23	0.003**
With	15	26	
AFP (μg/l)			
<200	6	28	0.3623
≥200	7	18	
Vascular invasion			
Without	9	13	0.0376*
With	7	36	
HBV DNA (cps/ml)			
<1000	3	14	>0.9999
≥1000	4	21	
Liver cirrhosis			
Without	7	24	0.7659
With	8	20	

*AFP* alpha-fetoprotein, *HBV* hepatitis B virus.

*p < 0.05; **p < 0.01.

Taken together, these findings indicate that RNF31 may function as an oncogenic molecule to promote HCC metastasis.

### RNF31 promotes the migration, invasion, and proliferation of HCC cells in vitro and in vivo

**Fig. 2:**
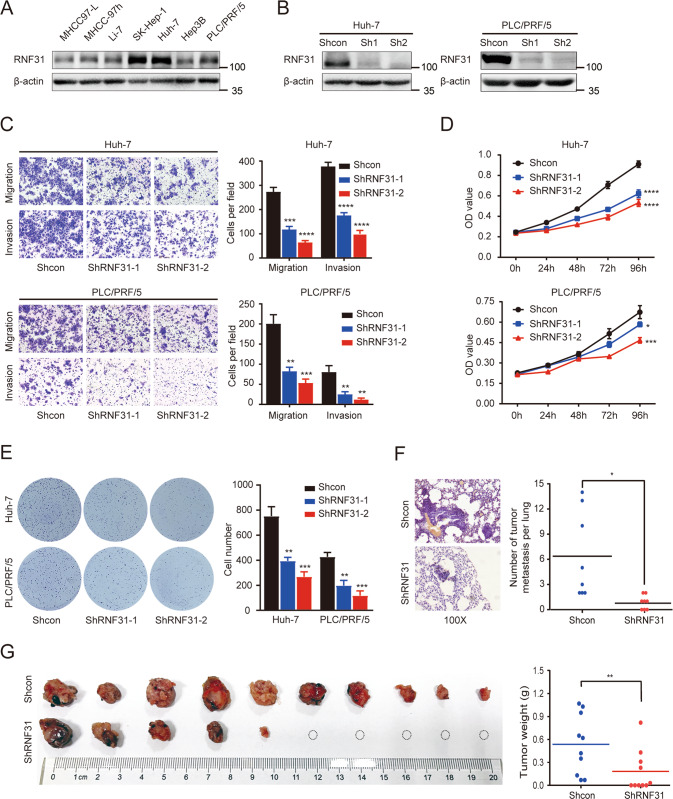
Down-regulation of RNF31 inhibits the migration, invasion, and proliferation of HCC cells in vitro and in vivo. **A** RNF31 protein levels were determined in seven HCC cell lines by performing a western bolt. **B** RNF31 knockdown efficacy was confirmed by western blot. **C** Representative images of migration and invasion assays for the RNF31 knockdown and the control HCC cells. The cells were counted under the microscope at ×100 magnification in five randomly selected single fields of vision. **D** CCK-8 assay was performed to investigate the effect of RNF31 knockdown on proliferation of PLC/PRF/5 and huh-7 cells. **E** Clone formation assay was performed to assess the clone formation abilities of the control and the RNF31 knockdown HCC cells. **F** Typical images of HE staining of pulmonary metastases. Lung tumor metastasis in mouse models was established by tail vein injection of control and *RNF31*-silenced huh-7 cells. **G** Effect of stable RNF31 knockdown on huh-7 cells growth in vivo by measuring the tumor weight. (*p < 0.05, **p < 0.01, ***p < 0.001, ****p < 0.0001).

We investigated the functional significance of RNF31 in HCC. First, RNF31 protein levels were determined in seven HCC cell lines (Fig. 2A). Considering the endogenous expression of RNF31 and transfection efficacy, RNF31 was knocked down using two shRNAs targeting two independent sites within RNF31 in HCC cells (Fig. 2B). Transwell assays revealed that RNF31 downregulation markedly suppressed HCC cell migration and invasion compared to the controls (Fig. 2C). The CCK8 assay (Fig. 2D) and colony formation analysis (Fig. 2E) revealed that knockdown of *RNF31* in HCC cells decreased their proliferation.

To confirm the oncogenic role of RNF31 in vivo, lung tumor metastasis in mouse models was established by tail vein injection of control and RNF31-silenced huh-7 cells. Mice injected with *RNF31*-silenced huh-7 cells exhibited fewer volumes and numbers of pulmonary metastases than mice injected with control cells (Fig. 2F).

Moreover, huh-7 cells with or without *RNF31*-knockdown were subcutaneously inoculated into the right and left flanks of nude mice. The tumors derived from *RNF31* stable-knockdown huh-7 cells were smaller than those derived from control tumors (Fig. 2G).

Thus, these results demonstrated a close association between high RNF31 expression and metastasis and growth of HCC cells.

### The RNF31 inhibitor gliotoxin inhibits the malignant behavior of HCC cells

**Fig. 3:**
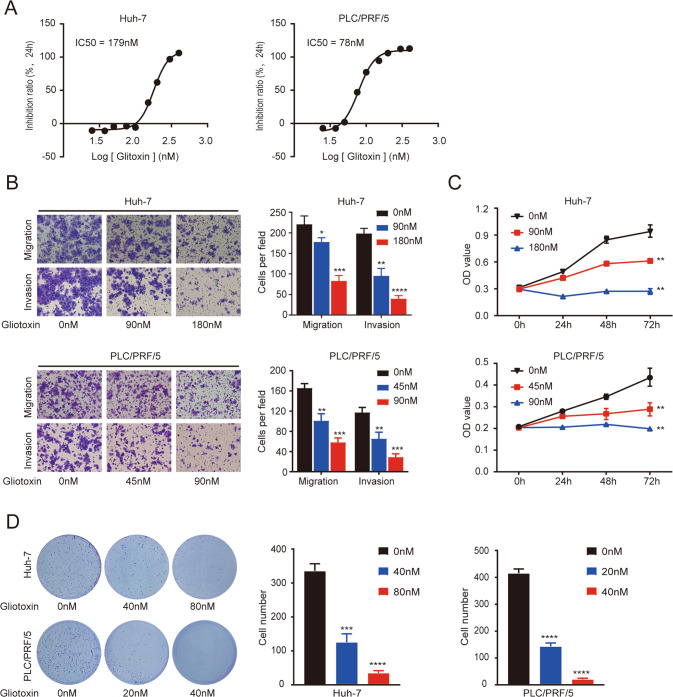
The RNF31 inhibitor gliotoxin inhibits the malignant behavior of HCC cells. **A** IC_50_ values of gliotoxin at 24 h in PLC/PRF/5 and huh-7 cells. IC_50_ was calculated using GraphPad Prism 8. **B** HCC cells were incubated with varying concentrations of gliotoxin for 24 h. Migration and invasion capacities of HCC cells were measured by transwell assays. Representative images of migration and invasion transwell assays are shown in the right panel and the transwell assay’s statistical results are shown in the left panel. **C** The CCK-8 assay was conducted to detect the cytotoxicity of different concentrations of Gliotoxin to the proliferative capacity of PLC/PRF/5 and huh-7 cells. **D** The cytotoxicity of various concentrations of Gliotoxin to the proliferative capacity of PLC/PRF/5 and huh-7 cells was detected via colony formation assay. (*p < 0.05, **p < 0.01, ***p < 0.001, ****p < 0.0001).

Gliotoxins are secondary metabolites produced by several species of fungi and have been found to inhibit RNF31 activity [30]. First, we observed the half-maximal inhibitory concentration (IC_50_) of gliotoxin in huh-7 (IC_50_ = 179 nM) and PLC/PRF/5 cells (IC_50_ = 78 nM) (Fig. 3A). HCC cells were then incubated with varying concentrations of gliotoxin. Transwell assays revealed that gliotoxin treatment decreased the migration and invasion capacities of HCC cells (Fig. 3B). Furthermore, CCK-8 and colony formation assays revealed that the proliferative capacity of PLC/PRF/5 and huh-7 cells was markedly reduced after 2 days of treatment with various concentrations of gliotoxin as compared with vehicle-incubated cells (Fig. 3C, D).

Together, our data revealed that a pharmacological inhibitor of RNF31 had an anti-HCC therapeutic effect.

### RBCK1 inhibits the ubiquitination-mediated degradation of RNF31 in HCC cells

**Fig. 4:**
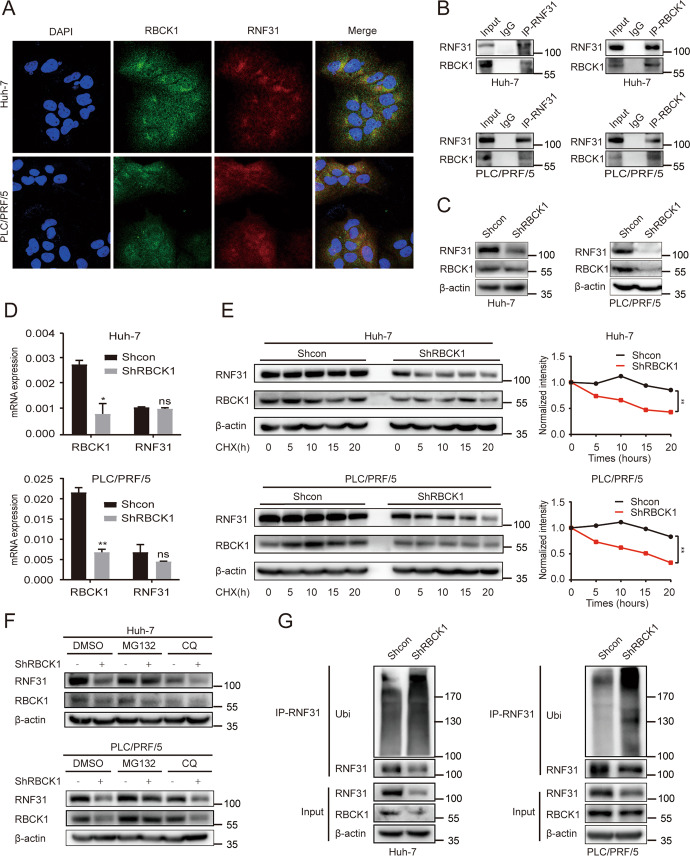
RBCK1 inhibits the ubiquitination-mediated degradation of RNF31 in HCC cells. **A** Immunofluorescence co-staining of RBCK1 and RNF31 in wild-type PLC/PRF/5 and huh-7 cells to detect colocalization. **B** Co-immunoprecipitation of endogenous RBCK1 and RNF31 in wild-type PLC/PRF/5 and huh-7 cells. **C** RNF31 protein level after the knockdown of RBCK1 in PLC/PRF/5 and huh-7 cells was analyzed by western blot. **D** *RNF31* mRNA level after RBCK1 knockdown in PLC/PRF/5 and huh-7 cells was determined by qPCR. **E** The control and RBCK1 knockdown PLC/PRF/5 and huh-7 cells were treated with CHX (20 μg/mL) at different time points. Whole-cell lysates were prepared and subjected to immunoblotting (left panel), and the RNF31 expression was quantified using Quantity One software (right panel). **p < 0.01. **F** The control and RBCK1 knockdown PLC/PRF/5 and huh-7 cells were pre-treated with the DMSO, proteasome inhibitor, MG132(10 μM), or the lysosome inhibitor, chloroquine (CQ, 25 μM) for 10 h, and then RBCK1, RNF31, and β-actin were detected via western blot. **G** The control and RBCK1 knockdown PLC/PRF/5 and huh-7 cells were incubated with MG132 at 10 μM for 10 h. Then cell lysates were prepared and immunoprecipitated with anti-RNF31 antibody and then immunoblotted with the indicated antibodies.

We explored the underlying upstream mechanisms of RNF31 overexpression in HCC. RBCK1 is a component of the LUBAC. Previous studies have indicated that RBCK1 functions as a pro-oncogenic factor and plays a crucial role in chemoresistance and stemness [25], growth ability, protein stability, and migration ability [28]. RBCK1 can bind RNF31 to form a complex exhibiting ubiquitin polymerization activity [13]. However, to date, it is unclear whether RBCK1 is involved in the development of HCC and how RBCK1 regulates the stability of RNF31. Immunofluorescence analysis showed the colocalization of RBCK1 and RNF31 (Fig. 4A), and a co-immunoprecipitation assay experiment confirmed the RBCK1/RNF31 interaction in HCC cells (Fig. 4B).

To verify that RBCK1 regulates RNF31, protein expression was detected in HCC cells transfected with shRBCK1. The results demonstrated that RNF31 protein levels were downregulated in HCC cells after the knockdown of *RBCK1* (Fig. 4C). In contrast, we found no marked change in the protein expression of RBCK1 after the knockdown of RNF31, suggesting that the protein regulation of the RBCK1‒RNF31 axis may be unidirectional (Supplementary Fig. 1). *RBCK1* knockdown did not alter the mRNA expression levels of *RNF31* (Fig. 4D), indicating that RBCK1 might regulate the degradation of RNF31 protein.

Next, we measured the effect of *RBCK1* knockdown on endogenous RNF31 protein stability by carrying out a CHX pulse-chase assay and found that *RBCK1* knockdown increased the degradation of RNF31 protein in both PLC/PRF/5 and huh-7 cells (Fig. 4E). The protein is degraded by proteasomes or lysosomes.

To investigate the mechanism responsible for RNF31 degradation, HCC cells were treated with the proteasome inhibitor MG132 and lysosomal inhibitor chloroquine (CQ). As expected, MG132, but not CQ, rescued RBCK1-mediated RNF31 downregulation (Fig. 4F).

Moreover, in vivo ubiquitination assays were performed to determine whether RBCK1 was involved in regulating RNF31 ubiquitination levels. The results indicated that the ubiquitination level of RNF31 was significantly increased by *RBCK1* knockdown in HCC cells (Fig. 4G).

Collectively, these results demonstrated that *RBCK1* deletion accelerates RNF31 degradation via the ubiquitin-proteasome pathway in HCC cells.

### *RBCK1* knockdown inhibits the migration, invasion, and proliferation of HCC cells

**Fig. 5:**
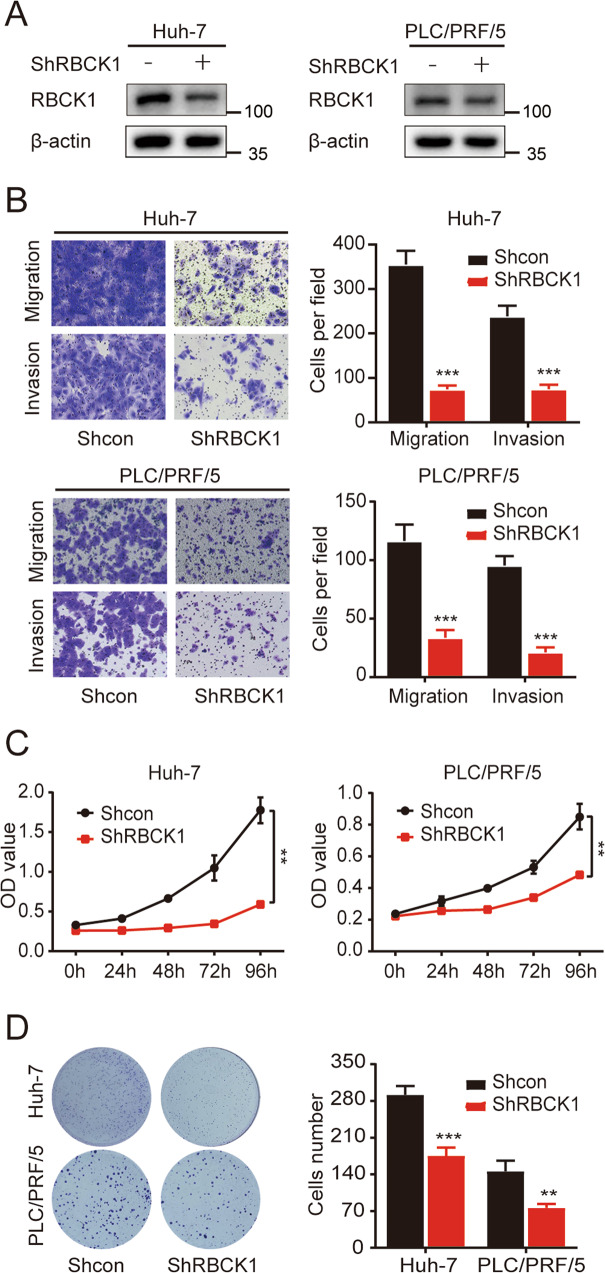
RBCK1 knockdown inhibits the migration, invasion, and proliferation of HCC cells. **A** Knockdown efficiency of RBCK1 in PLC/PRF/5 and huh-7 cells was confirmed by western blot. **B** Representative images of transwell migration and invasion assays for the RBCK1 knockdown and control HCC cells. The cells were counted under the microscope at ×100 magnification in five randomly selected single fields of vision. **C** CCK-8 assay was conducted to evaluate the effect of RBCK1 knockdown on PLC/PRF/5 and huh-7 cells proliferation. **D** Clone formation capacities of the control and RBCK1 knockdown PLC/PRF/5 and huh-7 cells were assessed by the clone formation assay. (**p < 0.01, ***p < 0.001).

Based on the finding that RBCK1 positively regulates RNF31, we probed the specific functional pattern of RBCK1 in HCC cells. As validated by western blotting, RBCK1 was silenced in PLC/PRF/5 and huh-7 cells (Fig. 5A). Transwell assays were performed to assess the invasion and migration abilities of HCC cells following RBCK1 depletion. Downregulation of RBCK1 significantly attenuated the migratory and invasive abilities of the HCC cells (Fig. 5B). In addition, CCK8 and colony formation assays suggested that *RBCK1* knockdown significantly reduced the growth of HCC cells (Fig. 5C, D).

Taken together, these results indicate that RBCK1 acts as an oncogene that facilitates HCC progression.

### RBCK1 exerts oncogenic effects partially through RNF31 regulation

**Fig. 6:**
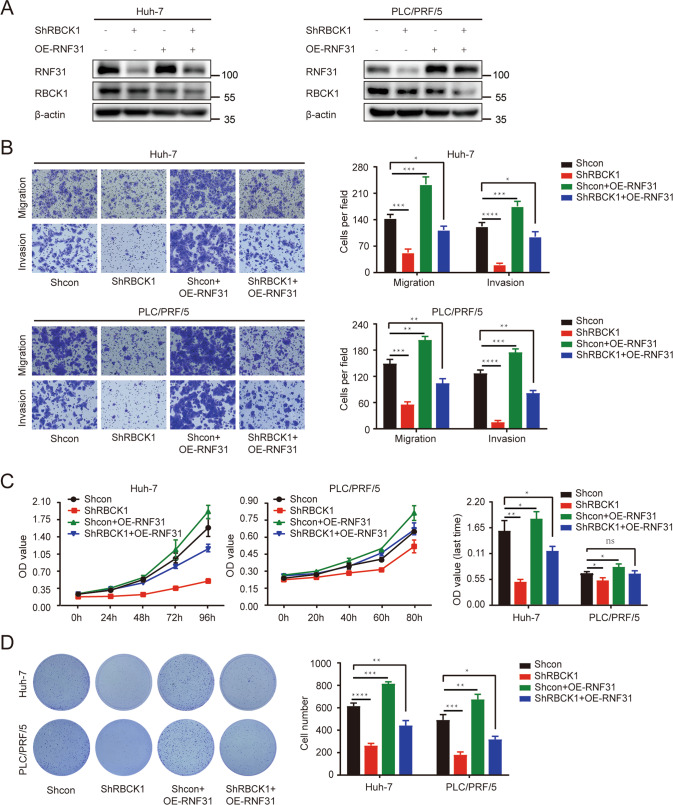
RBCK1 exerts oncogenic effects partially through regulating RNF31. **A** The efficacy of reintroducing sh*RBCK1* into HCC cells that stably overexpressed exogenous RNF31 was evaluated via western blot. **B** RNF31 impaired the HCC cell’s inhibition of migration and invasion induced by *RBCK1* knockdown in PLC/PRF/5 and huh-7 cells as confirmed by migration and invasion assays. **C** RNF31 abolished the proliferation inhibition of HCC cells imposed by *RBCK1* knockdown in PLC/PRF/5 and huh-7 cells as confirmed by CCK-8 assays. **D** RNF31 abolished the HCC cell’s suppression of proliferation caused by *RBCK1* knockdown in PLC/PRF/5 and huh-7 cells as confirmed by a colony formation assay. (*p < 0.05, **p < 0.01, ***p < 0.001, ****p < 0.0001).

We further investigated the biological significance of the RBCK1‒RNF31 axis in the metastasis and proliferation of PLC/PRF/5 and huh-7 cells. We conducted rescue experiments by restoring *RNF31* expression in HCC cells following *RBCK1* knockdown (Fig. 6A). Overexpression of *RNF31* partially rescued the suppression of HCC cell migration, invasion, and proliferation by *RBCK1* knockdown (Fig. 6B‒D).

Thus, these data revealed that RBCK1 acts upstream of RNF31 to regulate HCC progression.

### RBCK1 expression positively correlates with RNF31 expression in HCC tissues

**Fig. 7:**
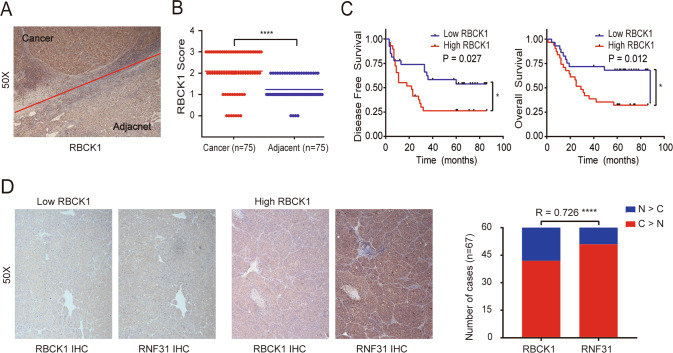
RBCK1 expression was positively correlated with RNF31 expression in HCC tissues. **A** Typical IHC picture of RBCK1 in paired HCC and corresponding noncancerous liver tissues. **B** IHC assay was performed to detect RBCK1 expression levels in HCC tissues and adjacent noncancerous liver tissues. Immunohistochemical score were analyzed using Wilcoxon’s test (*n* = 75, ****p < 0.001). **C** Overall survival and tumor-free survival of HCC patients were analyzed by Kaplan–Meier survival analysis (log-rank test, *p < 0.05). IHC staining was applied to divide the HCC patients into the high and low expression groups of RBCK1. **D** Representative images of immunohistochemical staining of RNF31 in HCC tissues with different expression levels of RBCK1. Correlation between RBCK1 and RNF31 protein levels in HCC tissues was analyzed by Spearman correlation analysis (*n* = 67, *R* = 0.726, ****p < 0.001).

Based on our findings, RBCK1 acts upstream of RNF31 to regulate HCC progression. To reveal the clinical implications of RBCK1 expression in HCC, we first evaluated RBCK1 expression in TCGA online database. *RBCK1* mRNA was highly expressed in liver cancer samples (Supplementary Fig. 2A). High *RBCK1* mRNA expression was associated with shorter overall survival (Supplementary Fig. 2B). Consistently, these results were also confirmed by IHC in HCC samples, which demonstrated that the expression of RBCK1 was significantly higher in HCC than in the adjacent normal tissues (Fig. 7A, B) and that higher IHC scores of RBCK1 were positively associated with shorter tumor-free survival and overall survival (Fig. 7C).

To clarify the clinical relevance of RBCK1 and RNF31 protein expression levels, consecutive paraffin sections of HCC tissue were used for IHC scoring. RNF31 expression levels were generally elevated in HCC samples with high expression levels of RBCK1. Spearman’s correlation analysis revealed a significant correlation between RBCK1 and RNF31 expression in 67 HCC tissues (*p* < 0.0001, *R* = 0.726; Fig. 7D).

### Exploration of potential downstream targets of RNF31 in HCC

RNF31 has been reported to regulate linear ubiquitination modifications of proteins. To reveal the potential downstream targets of RNF31 in HCC cells, we identified RNF31-interacting proteins in HCC cells after cells were treated with the proteasome inhibitor MG132 for 10 h by using immunoprecipitation and mass spectrometry (IP-MS). In this way, 477 RNF31-interacting proteins were identified in huh7 cells, and 459 RNF31-interacting proteins were identified in PLC/PRF/5 cells, of which 434 proteins were identical (Supplementary Fig. 3A). Moreover, mass spectrometry (Supplementary Fig. 3B) and mass spectrometry-based quantification of ubiquitin chains (Supplementary Fig. 3C) were used after the *RNF31* knockdown.

Based on the results described above, combined with the literature, P53, PGM1, FGB, AGXT, ESR1, ANXA2, EIF3F, RTN3, PRDX4, HSP90AA1, and SEPT2 were selected for further research. Western blot analysis suggested no significant changes in the protein levels of these molecules after *RNF31* knockdown (Supplementary Fig. 3D). Therefore, additional studies are needed to elucidate the downstream molecular mechanisms of RNF31.

## Discussion

The present study revealed that RBCK1 and RNF31 may be involved in the metastasis and growth of HCC and may serve as indicators of poor prognosis. Moreover, our research further uncovered a new mechanism by which RBCK1 may positively regulate the stability of the RNF31 protein through the ubiquitin–proteasome pathway. Finally, we found that gliotoxin, an inhibitor of RNF31, may suppress the malignant biology of HCC cells.

Analysis of HCC and non-cancer specimens from TCGA and from our HCC sample bank revealed that both RNF31and RBCK1 are elevated in HCC tissues. High expression of these genes was associated with a poor prognosis. Clinically, upregulated RNF31 expression was significantly associated with more satellite foci and vascular invasion in HCC patients. Moreover, previous studies have revealed a markedly negative correlation between RNF31 expression and survival time in a variety of cancer types, such as prostate [19], breast [21], gastric [16], and colon cancers [20]. In addition, RBCK1 is highly expressed in pulmonary adenocarcinoma [23], breast cancer [24], colon cancer [25], and renal cell carcinoma [26, 27]. These observations agree with our findings, suggesting that RNF31 and RBCK1 may be used as prognostic markers in patients with HCC.

RNF31 has previously been characterized as a pro-oncogenic factor in various tumors. For example, in breast cancer, RNF31 supports ERα mono-ubiquitination and enhances its stability, thereby promoting breast cancer proliferation [17]. Another study revealed that RNF31 promotes breast cancer development by promoting P53 ubiquitination and degradation [11], which has also been reported in renal cancer [11]. The findings of these studies are compatible with those of our study, revealing that the upregulation of RNF31 may act as a positively regulated effector of cancer. However, in a mouse model with liver parenchymal *RNF31* knockout, the liver parenchyma showed increased caspase activity, leading to increased apoptosis, liver regeneration, and DNA damage, ultimately resulting in liver tumorigenesis [31].

Furthermore, analogous phenomena have been observed in another study. YAP and TAZ can facilitate liver tumor growth but exhibit tumor suppressor activity in normal hepatocytes surrounding the liver tumor [32]. Collectively, these findings suggest that RNF31 may act as a tumor suppressor in the normal liver parenchyma.

Taken together, RNF31 may play different roles in the occurrence and development of HCC, and further studies are necessary to confirm this undetermined observation.

During the primary screen of RNF31 downstream candidate molecules, we did not find any changes in the protein expression levels of these candidate molecules (Supplementary Fig. 7D). One possible reason for these results is the presence of other downstream effectors. Another possible explanation is that RNF31 does not affect the protein expression level of these candidates but only affects the proteins’ functions by post-translational modifications, such as phosphorylation, methylation, and other modifications. For example, UHRF1 is an E3 ubiquitin ligase that regulates substrate ubiquitination and methylated-DNA binding functions [33]. Interestingly, RNF31 has been shown to regulate P53 protein levels in many tumors [11, 20]. However, the protein level of P53 did not change after the knockdown of *RNF31* in PLC/PRF/5 and huh-7 cells, in which P53 is mutated. After mass spectrometry-based quantification of ubiquitin chains after *RNF31* knockdown, we noted that both KEGG pathway analysis and Gene Ontology enrichment analysis suggested that RNF31 might be associated with metabolic pathways; moreover, it has been reported that LUBAC is involved in inflammation, NF-κB signaling, apoptosis, and mTOR regulation [34], suggesting potential directions for future work.

The role of RBCK1 in HCC and the mechanisms of interaction between RBCK1 and RNF31 remain unclear. Here, we revealed that *RBCK1*-knockdown markedly reduces the malignant activity of HCC. Similarly, RBCK1 also exhibits cancer-promoting capabilities in renal cell cancer [26], colorectal cancer [25], breast cancer cells [35, 36], and lung tumors [23]. Taken together, RBCK1 may be a pro-tumorigenic target. Mechanistically, RBCK1 ubiquitinates PKCζ and promotes its degradation in lung tumors. Silencing of RBCK1 impairs lung tumor growth [23]. In addition, RBCK1 regulates cell cycle progression and growth in ERα-positive breast cancer cells by facilitating the transcription of ERα and cell cycle protein B1 [35].

Here, we identified a novel potential mechanism by which RBCK1 may positively mediate the protein stability of RNF31. Knockdown of *RBCK1* downregulated the protein level of RNF31 without affecting its mRNA expression levels. More specifically, *RBCK1* knockdown strikingly accelerated the degradation of the RNF31 protein through the proteasome pathway, while the ubiquitination level of RNF31 also increased after the depletion of RBCK1. Finally, we elucidated the role of the RBCK1‒RNF31 axis in HCC. According to the previous studies, RBCK1 directly binds to RNF31 and promotes RNF31 protein stability [37, 38]. These findings may provide a mechanistic basis for the development of HCC. However, the precise molecular mechanism by which RBCK1 regulates the ubiquitination of RNF31 warrants further investigation.

Various proteasome inhibitors, such as bortezomib and carfilzomib, have been widely used in the clinical treatment of non-solid tumors. However, proteasome inhibitors have failed to show any efficacy in patients with solid tumors in clinical studies [33]. Therefore, the development of more proteasome inhibitors may provide additional insights into the treatment of solid tumors. To the best of our knowledge, no pharmacological inhibitor of RNF31 is currently used in clinical treatment. Further research is underway to investigate RNF31 inhibitors. For example, inhibitors targeting the active site of the RNF31 subunit have been studied by covalent ligand screening of the corresponding fragment [39]. Another study selected single-domain antibodies based on human scaffolds to recognize the catalytic domain of RNF31, thereby providing a robust reference for ligands targeting a cysteine at the active site of RNF31 [40]. In addition, gliotoxin has been reported to inhibit RNF31 activity [30]. In our study, the malignant phenotype of HCC cells was suppressed by gliotoxins, suggesting that RNF31 may serve as a therapeutic target in HCC. However, gliotoxin has multiple targets other than RNF31 and has many side effects in clinical use [30]. Therefore, more target-specific inhibitors of RNF31 should be developed in the future.

In conclusion, our study suggested the potential roles and related novel mechanisms of RBCK1 and RNF31 in HCC. These results provide insight into potential direct prognostic markers as well as therapeutic possibilities for HCC treatment.

## Materials and methods

### Specimen collection

For experiments on human HCC tissue samples, all tissues were collected from the Biospecimen Bank for Liver Cancer, Zhongshan Hospital, Xiamen University. Human HCC tissue specimens were collected between 2013 and 2018. None of the patients were diagnosed with other cancer types or had received anticancer therapy before surgical resection. Tissue specimens were collected, flash-frozen in liquid nitrogen, and immediately stored at −80 °C for subsequent experiments. Follow-up information was collected using telephone interviews and by reviewing the patients’ medical records.

All participants signed an informed consent form. All informed consent forms and experiments were approved by the Ethics Committee of Zhongshan Hospital, Xiamen University, and were in accordance with the Declaration of Helsinki.

### Hematoxylin‒eosin and immunohistochemistry staining

Specimens were formalin-fixed and paraffin-embedded. Paraffin sections (4.5-μm thick) were then de-paraffinized using xylene, dehydrated in graded alcohol, and washed three times with phosphate-buffered saline. Paraffin sections were stained with hematoxylin and eosin (HE) for light microscopy. For immunohistochemistry (IHC), antigen retrieval was performed using boiling sodium citrate buffer. The sections were incubated with endogenous peroxidase blocking reagent, a primary antibody, a primary antibody enhancer, biotinylated secondary antibodies, and diaminobenzidine. Two independent pathologists classified the immunostained sections and reported the mean scores. A senior pathologist reclassified these inconsistent scores. Five random 200× microscopic fields from each slide were evaluated, and the staining scores were classified as follows: 0, no staining; 1, light yellow; 2, intermediate positive; and 3, tawny. Almost all cells were positively stained with RNF31 and RBCK1; therefore, we classified the protein levels into two groups based on the staining intensity: a score ≤2 was considered “low expression” and that >2 was treated as “high expression.” All reagents were purchased from Maixin (Fuzhou Biotechnology, Fujian, China), except for the primary antibodies.

Other materials and methods in detail are provided in the Supplementary Information.

## Supplementary information

Supplementary Information

Original Data File

## Data Availability

The datasets used during this research are available. The datasets used and/or analyzed during the current study are available from the corresponding author on reasonable request.
