# Supplementary material for: RBCK1 promotes hepatocellular carcinoma metastasis and growth by stabilizing RNF31
Source: Cell Death Discov. 2022 Jul 22;8:334. doi: 10.1038/s41420-022-01126-x (PMC9307510; doi:10.1038/s41420-022-01126-x)
Supplement: Supplementary file 1 — Supplementary Information [file 41420_2022_1126_MOESM1_ESM.docx]

**Supplementary Information**

**RBCK1 promotes hepatocellular carcinoma metastasis and growth by stabilizing RNF31**

Content:

1. Supplementary materials and methods

2. Supplementary Table 1

3. Supplementary Figures and Figure Legends

# Supplementary materials and methods

***Cell culture***

Huh-7, HEK293T, SK-Hep-1, MHCC97-L, MHCC-97h, Li7, Hep3B, and PLC/PRF/5 cells were purchased from the Cellcook Biotechnology Company (Guangzhou, China). All cell lines were confirmed by short tandem repeat detection. All cells were grown in humidified 5% CO_2_ incubators at 37°C in Dulbecco’s modified Eagle’s medium (DMEM; Biological Industries, Beit Haemek, Israel) containing 1% penicillin/streptomycin (Millipore, Billerica, MA, USA), to which 10% fetal bovine serum (FBS) was added (Biological Industries).

***RNA extraction and quantitative real-time PCR***

An Eastep Super Total RNA Extraction Kit (Promega, Madison, WI, USA) was used for total RNA extraction. cDNA was synthesized using an Evo M-MLV RT Kit (Accurate Biotechnology, Shanghai, China). Quantitative real-time polymerase chain reaction (qPCR) was performed using the SBYR Green Master Mix (Thermo Fisher Scientific, Waltham, MA, USA) in a Light-Cycler 96 PCR detection system (Roche Applied Sciences, Penzberg, Germany). The primer sequences used were as follows:

RNF31-forward: 5'-GAGCCCCGAAACTACCTCAAC-3';

RNF31-reverse: 5'-CTTGACACCACGCCAGTACC-3';

RBCK1-forward: 5'-TGCTCAGATGCACACCGTC-3';

RBCK1-reverse: 5'-CAAGACTGGTGGGAAGCCATA-3';

GAPDH-forward: 5'-GGAGCGAGATCCCTCCAAAAT-3';

GAPDH-reverse: 5'-GGCTGTTGTCATACTTCTCATGG-3'.

***Plasmid constructs and transfection***

For RNF31 overexpression, complementary human DNAs corresponding to full-length RNF31 were cloned into the lentiviral vector PLV-puro (Addgene Inc., Watertown, MA, USA). For stable knockdown, short-hairpin RNA (shRNA) sequences were inserted into the PLV-shRNA-puro plasmid. The following shRNA sequences were used: RNF31-sh1, 5'-CCGAGATGT-GCTGCGATTATA-3'; RNF31-sh2, 5'-GCGTGGTGTCAAGTTTAATAA-3'; and BCK1-sh, 5'-AGATCGTGGTACAGAAGAA-3'. Lentivirus-expressing vectors and control plasmids were co-transfected with psPAX2 and pMD.2G into 293T cells. After 24 h, lentiviral particles were collected and were used to infect HCC cells for 48 h with 5 μg/mL polybrene (Solarbio, Beijing, China). Stably expressing cells were selected using 2 μg/mL puromycin (Solarbio).

***Western blot analysis***

Protein lysates were obtained from cells and tissues using cell lysis buffer (Beyotime Biotechnology, Shanghai, China) plus a 1× protease inhibitor cocktail (Sigma‒Aldrich, St Louis, MO, USA). Total protein concentration was calculated using a BCA kit (Thermo Fisher Scientific) according to the manufacturer’s instructions. The protein samples were then separated using 10% SDS-PAGE and transferred onto PVDF membranes (Millipore). The membranes were blocked with 5% milk in TBST for 50 min. After blocking, membranes were incubated with primary antibodies at 4°C overnight. The membranes were then washed and incubated with secondary antibodies at room temperature for at least 50 min. Finally, the membranes were visualized using Immobilon Western Chemiluminescence HRP Substrate (Solarbio). The antibodies used are listed in Table S1.

***Migration and invasion assays***

For both migration and invasion assays, HCC cells were trypsinized, resuspended in 200 μl DMEM, and added to the top chamber of a Transwell device. DMEM (550 μl DMEM supplemented with 10% FBS, was added to the lower chamber. After incubation, the inserts were removed, the medium was aspirated, and cells on the upper side of the insert were wiped off using cotton swabs. Migrating or invading cells were assessed via standard crystal violet staining, imaged, and counted at 20× magnification using a dissection microscope.

***Colony formation***

To assess colony formation, cells (2.5 × 10^3^) were added into 6-well plates and cultured for approximately 14 days. The cells were then fixed with 4% paraformaldehyde for 20 min and stained with 1.0% crystal violet for 13 min.

**Proliferation assays**

To detect cell proliferation, a Cell Counting Kit 8 (CCK8; Dojindo, Kumamoto, Japan) was used according to the manufacturer’s instructions. The fluorescence (450 nm) was measured at 450 nm.

***Co-immunoprecipitation assay***

A co-immunoprecipitation assay (co-IP) was carried out using a Co-Immunoprecipitation Kit (Thermo Fisher Scientific) following the manufacturer’s instructions. The HRP-conjugated Recombinant Rabbit Anti-Mouse IgG Kappa Light Chain (Proteintech, Wuhan, China, SA00001-19) were used to detect the RBCK1 primary antibodies (Abcam, ab219955).

***Protein half-life assays***

For protein half-life assays ^1^, cells were incubated with cycloheximide (CHX; 10 μg/ml) at different time points after transfection. Cells were lysed and analyzed by western blotting.

***In vivo ubiquitination assay***

For *in vivo* ubiquitination assays, the cells were treated with 10 μM MG-132 for 10 h. The cell lysate extracted from *RBCK1*-knockdown and control HCC cells was co-immunoprecipitated with an anti-RNF31 antibody, and the ubiquitination level of RNF31 was measured using an anti-ubiquitin antibody.

***Mass spectrometry***

The cell samples were subjected to mass spectrometry by NOVOGENE Co. Ltd. (Nanjing, China). Mass spectrometry was performed as previously described ^2^.

**Animal studies**

Male SCID mice and BALB/c nude mice were supplied by Shanghai SLAC Laboratory Animal CO Ltd. Permission for animal experiments was granted by the ethics committee of Xiamen University and was performed according to the Guide for Care and Use of Laboratory Animals.To investigate the effect of RNF31 on *in vivo* metastasis, 1.5 × 10^6^ huh-7 cells suspended in 0.1 ml serum-free culture medium were intravenously injected into SCID mice. The mice were euthanized 9 weeks later, and lung tissue metastatic lesions were confirmed by histological analysis. The tissue was fixed with 10% neutral formalin, and blocks of formalin-fixed paraffin-embedded tissue were cut into 4.5-μm-thick sections and stained with HE.

To explore the function of RNF31 in *in vivo* growth, 5 × 10^6^ huh-7-shCtrl and huh-7-ShRNF31 cells were inoculated subcutaneously into both sides of the backs of BALB/c nude mice. The mice were euthanized 4 weeks later, and subcutaneous tumors were collected and the tumor weight and volume compared.

***Statistical analysis***

The experimental data were presented as means ± standard error of the mean carried out using SPSS software (version 23.0; IBM Corp., Armonk, NY, USA). The Student’s *t*-test was used to compare the differences between two related samples. Fischer’s exact test was used to elucidate the relationship between the RNF31 score and clinicopathological factors in patients with HCC. Spearman's rank correlation was used to examine the correlation between RNF31 and RBCK1 expression levels. The correlation between protein expression levels and prognosis was estimated using the Kaplan‒Meier method and log-rank test. Statistical significance was set at < 0.05.

1. Lu K, Yin X, Weng T, Xi S, Li L, Xing G*, et al.* Targeting WW domains linker of HECT-type ubiquitin ligase Smurf1 for activation by CKIP-1. *Nat Cell Biol* 2008, **10**(8)**:** 994-1002.

2. Tan Y, Jin Y, Zhao P, Wu J, Ren Z. Lipid droplets contribute myogenic differentiation in C2C12 by promoting the remodeling of the acstin-filament. *Cell Death Dis* 2021, **12**(12)**:** 1102.

# 2. Supplementary Table 1

Table S1. Primary antibodies

| Application | Name | Code | Company |
| --- | --- | --- | --- |
| WB/IP | Anti-RNF31 | ab125189 | Abcam |
| IHC/IF | Anti-RNF31 | ab187976 | Abcam |
| WB | Anti-β-actin | 66009-1-Ig | Proteintech |
| WB | Anti-GAPDH | 60004-1-Ig | Proteintech |
| IF/IP | Anti-RBCK1 | sc-365523 | Santa Cruz |
| WB/IHC | Anti-RBCK1 | ab219955 | Abcam |
| WB | Anti-ubiquitin | 10201-2-AP | Proteintech |
| WB | Anti-P53 | A19585 | Abclonal |
| WB | Anti- PGM1 | 15161-1-AP | Proteintech |
| WB | Anti- FGB | 16747-1-AP | Proteintech |
| WB | Anti-AGXT | 22394-1-AP | Proteintech |
| WB | Anti- ESR1 | A12976 | Abclonal |
| WB | Anti- ANXA2 | 11256-1-AP | Proteintech |
| WB | Anti-UB | 10201-2-AP | Proteintech |

# 3. Supplementary Figures and Figure Legends


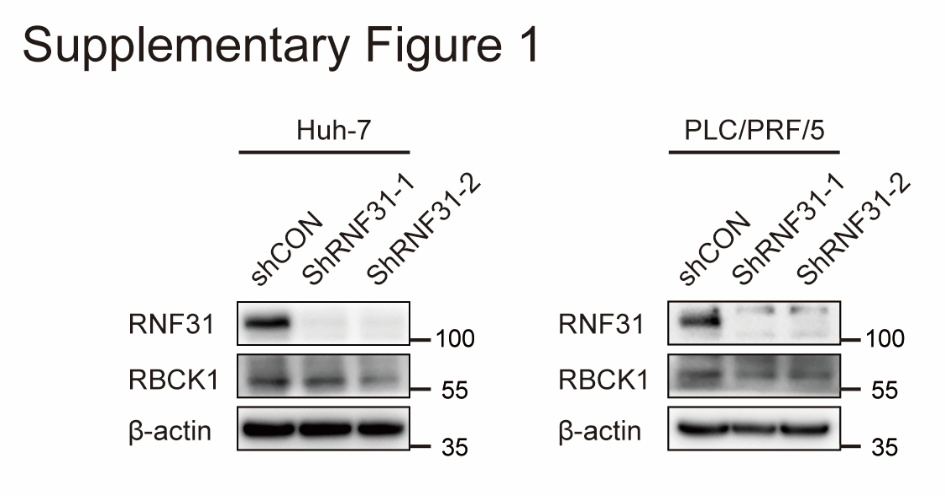


Supplementary figure 1. RBCK1 protein level upon RNF31 knockdown in PLC/PRF/5 and huh-7 cells was detected using western blot.


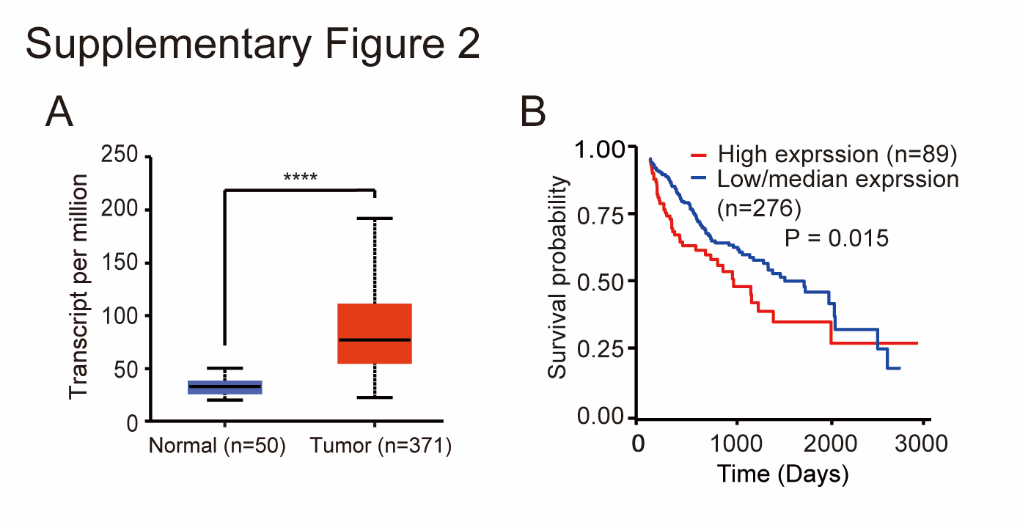


Supplementary figure 2 Relationship between RBCK1 expression and HCC patient’s prognosis was analyzed in the TCGA database.

A. RBCK1 expression in HCC samples and normal liver tissues from TCGA database. B. Kaplan-Meier analysis showed the overall survival in patients with low and high RBCK1 expression.


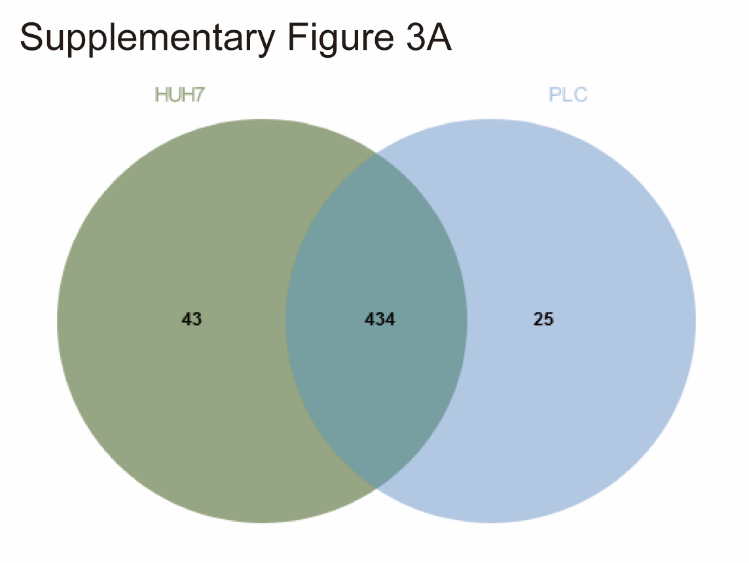


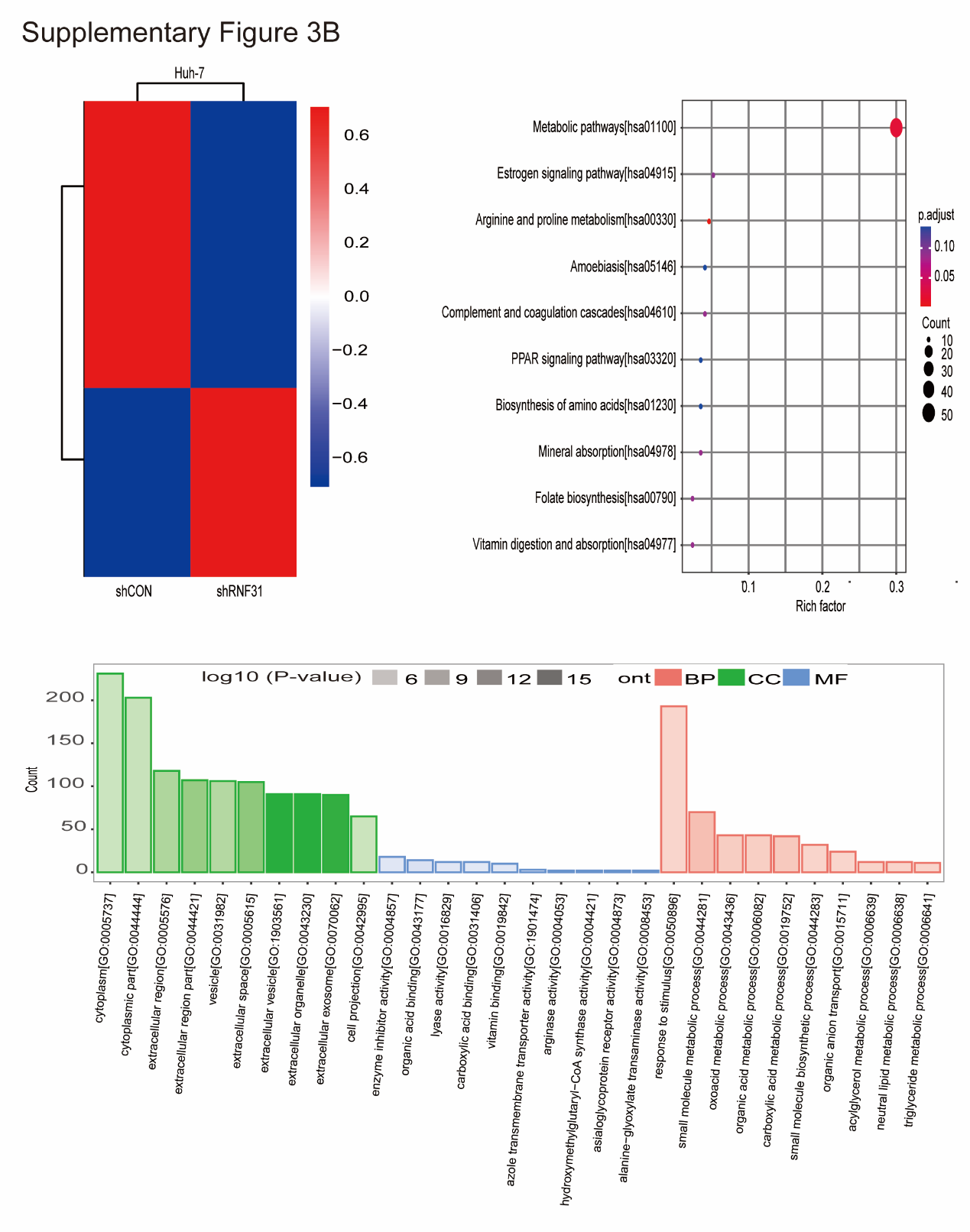

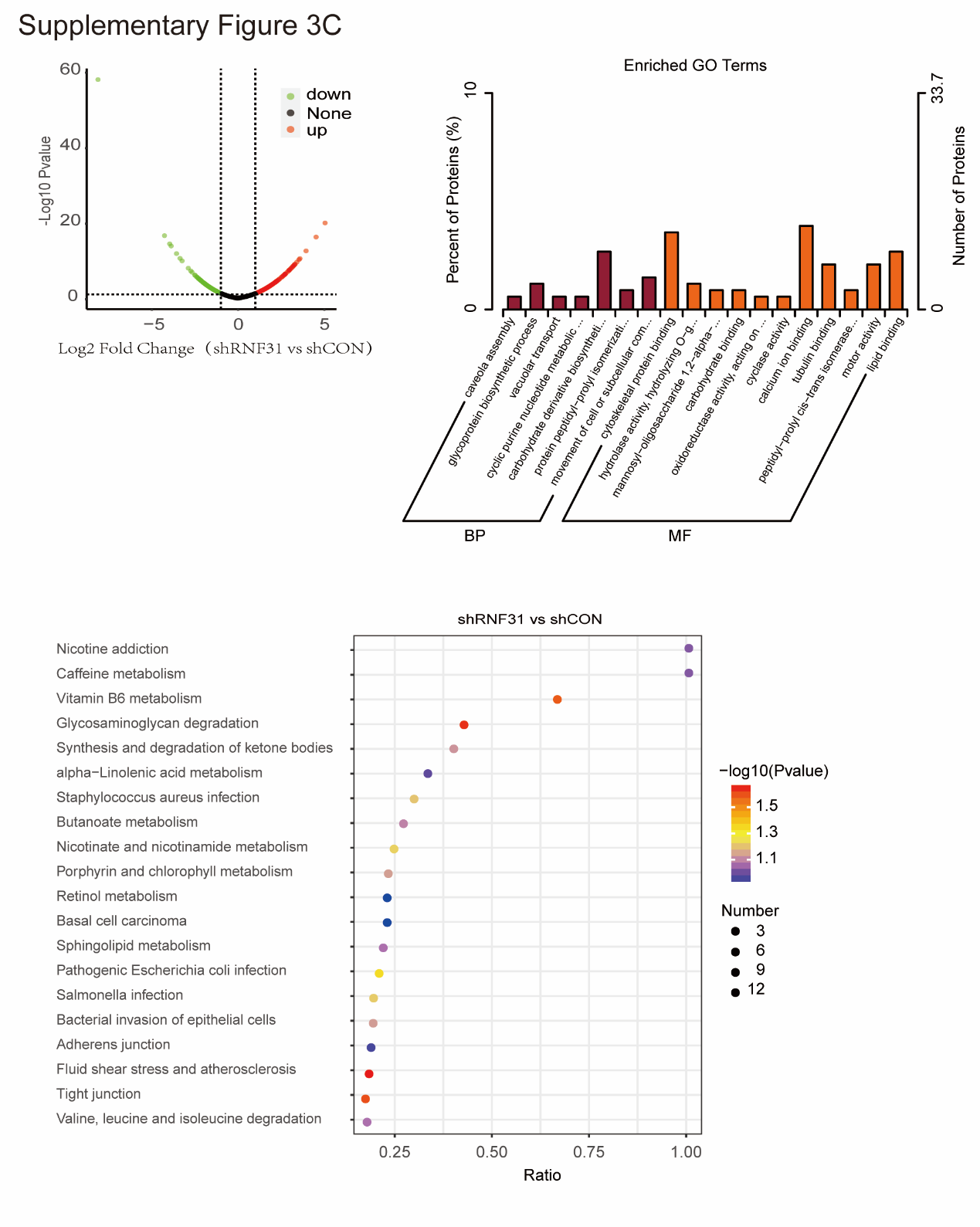

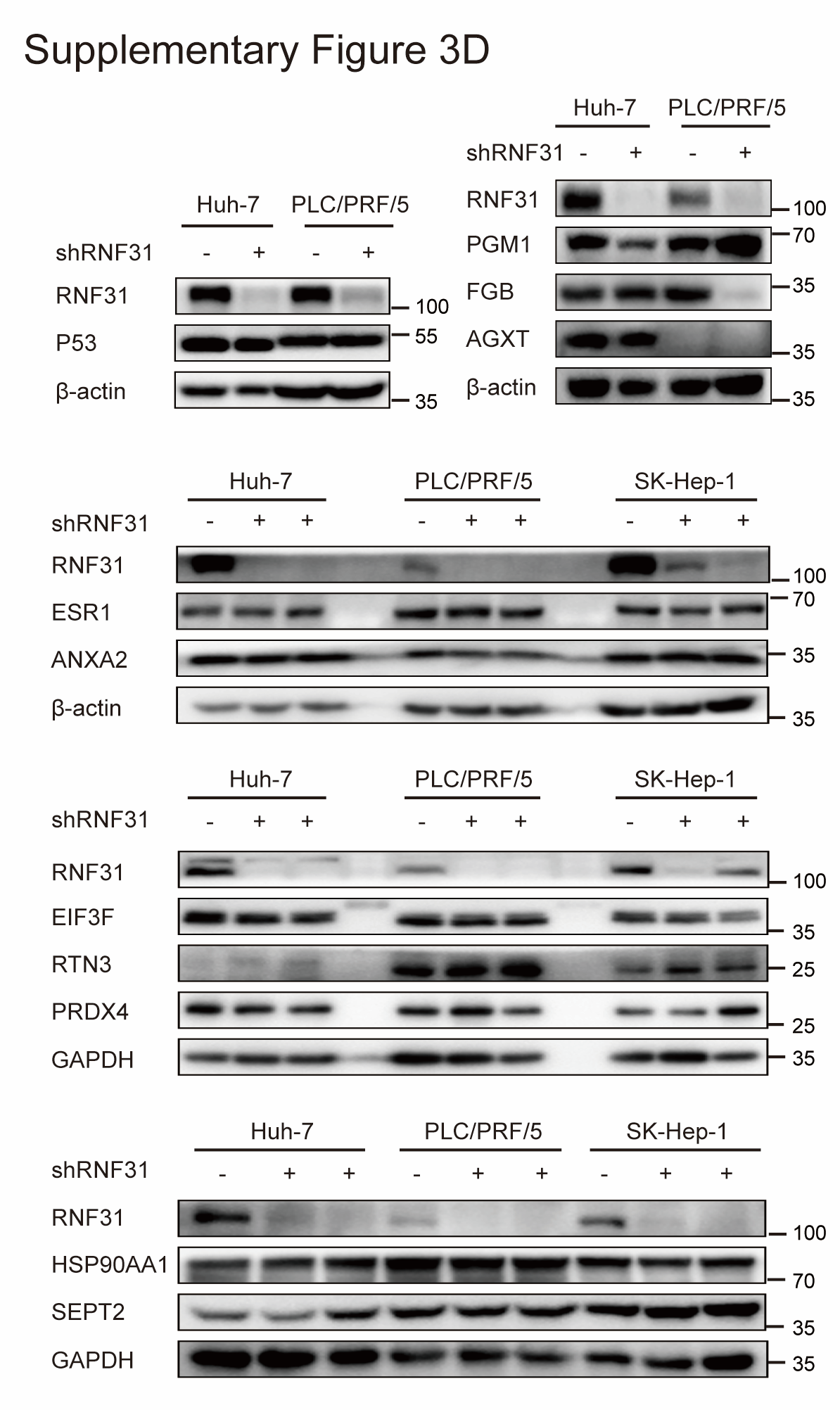


**Supplementary figure 3. The potential downstream targets of RNF31 were explored in HCC.**

A. Identification of RNF31 interacting proteins by performing immunoprecipitation and mass spectrometry (IP-MS) in PLC/PRF/5 and huh-7 cells. Venn diagram represents the unique and overlapping proteins identified in PLC/PRF/5 and huh-7 cells.

B. Mass spectrometry (MS) quantified was used to analyze PLC/PRF/5 and huh-7 cells after RNF31 knockdown.

C. Mass spectrometry-based quantification of ubiquitin chains was applied to analyze huh-7 cells after knockdown of RNF31.

D. Western-blot applied to validate the potential downstream proteins of RNF31.
