# Supplementary material for: RBCK1 promotes hepatocellular carcinoma metastasis and growth by stabilizing RNF31
Source: Cell Death Discov. 2022 Jul 22;8:334. doi: 10.1038/s41420-022-01126-x (PMC9307510; doi:10.1038/s41420-022-01126-x)

Uncropped western blot

Figure 1C

Tissues 1-3

GAPDH

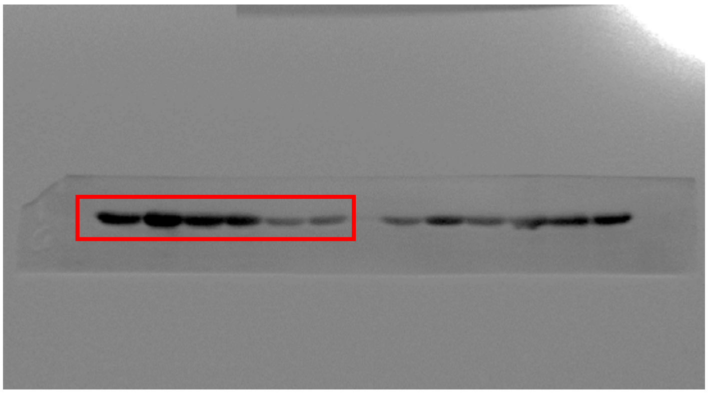

RNF31

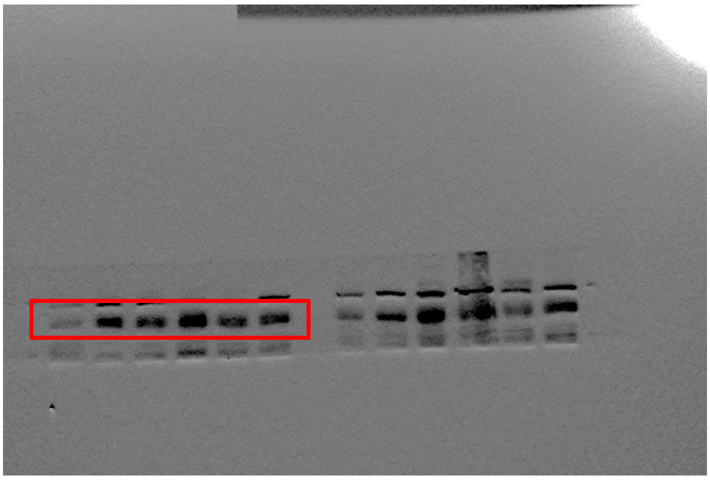

Tissues 4-6

GAPDH

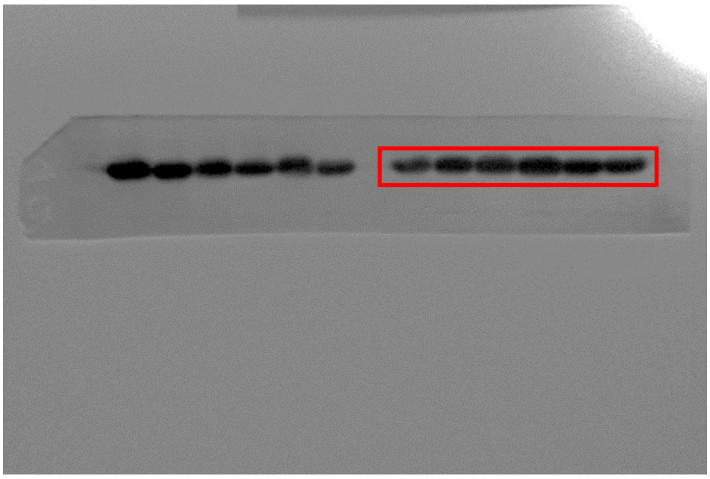

RNF31

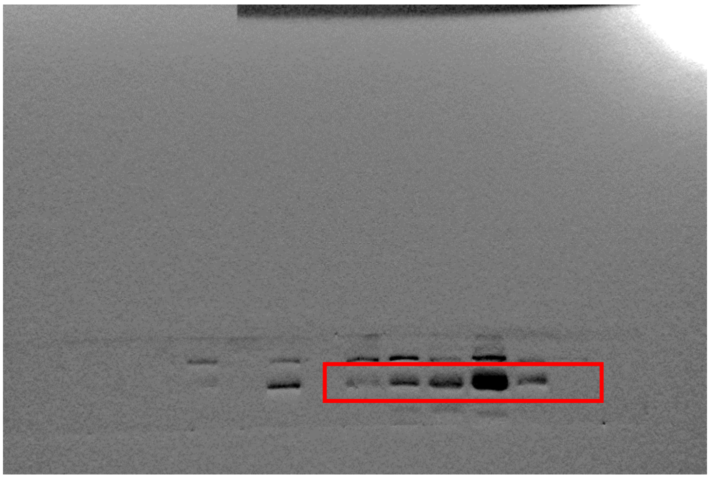

Tissues 7-9

GAPDH

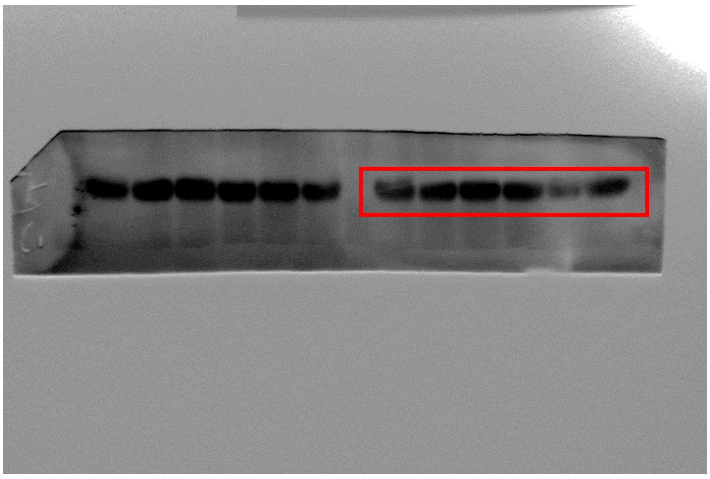

RNF31

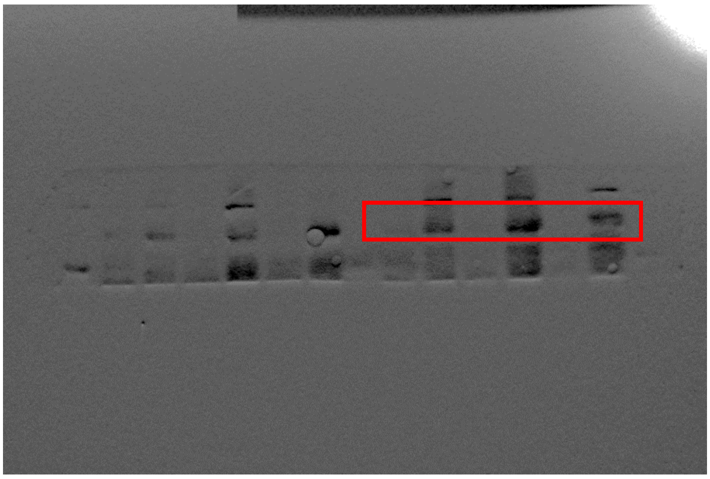

Tissues 10-12

GAPDH

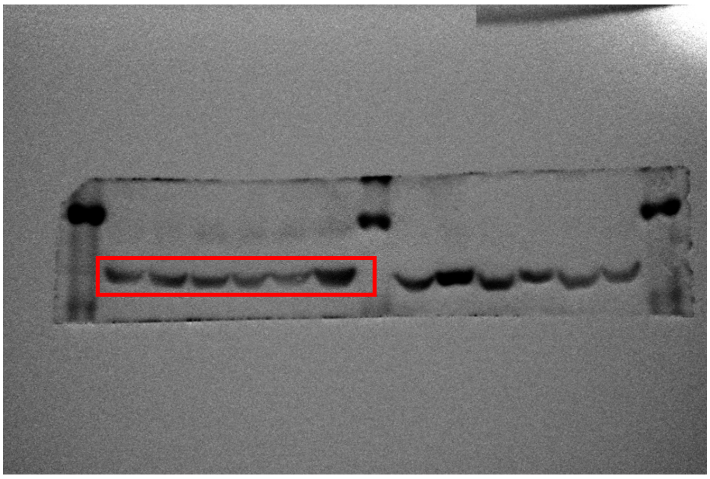

RNF31

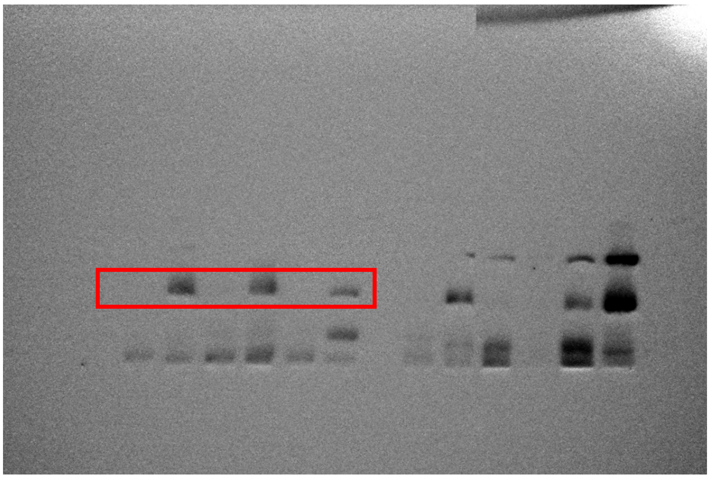

Tissues 13-15

GAPDH

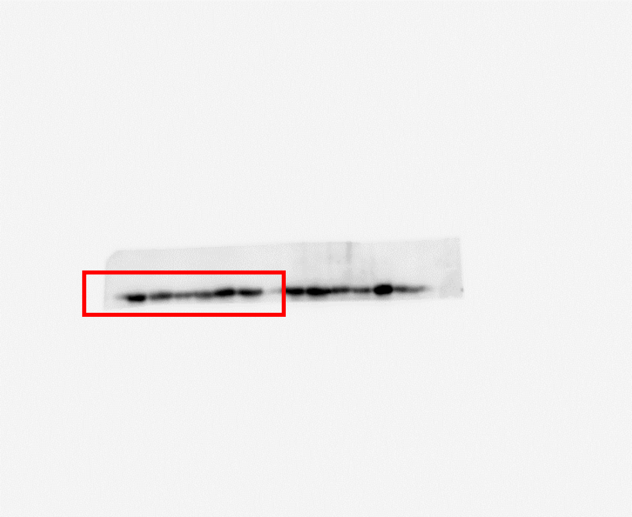

RNF31

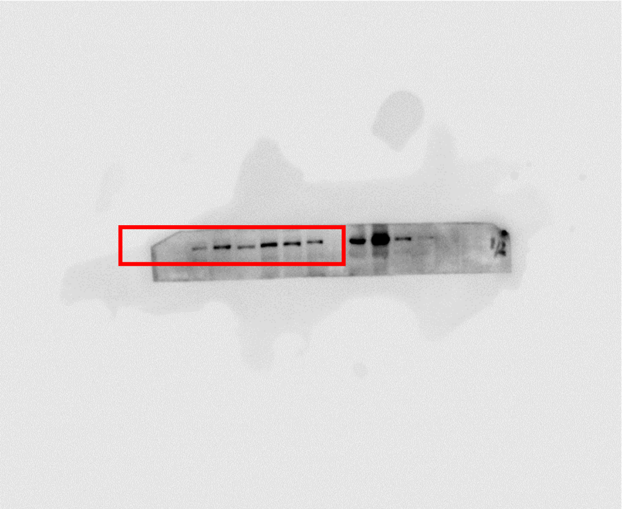

Figure 2A

ACTIN

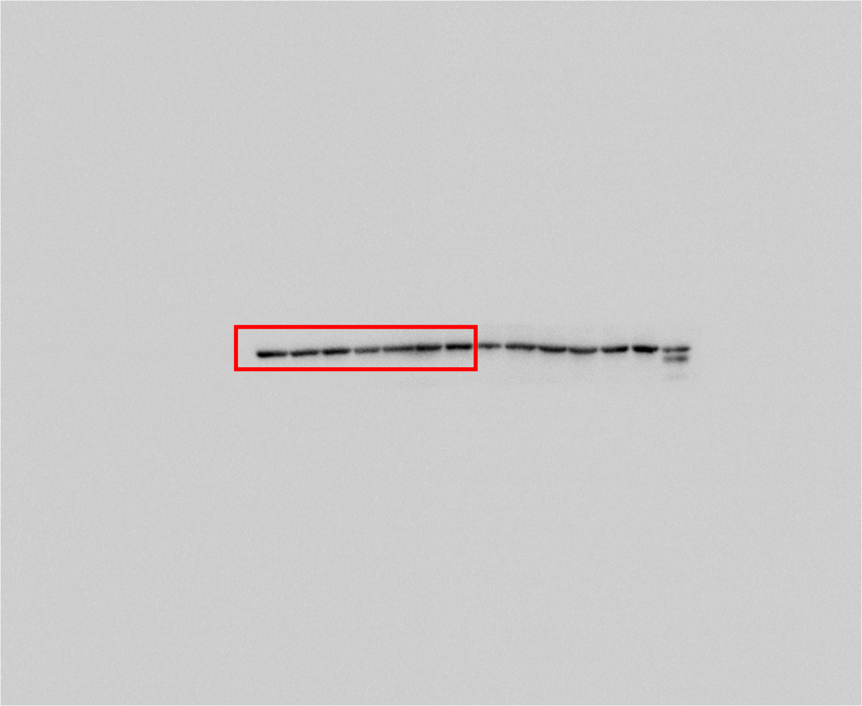

RNF31

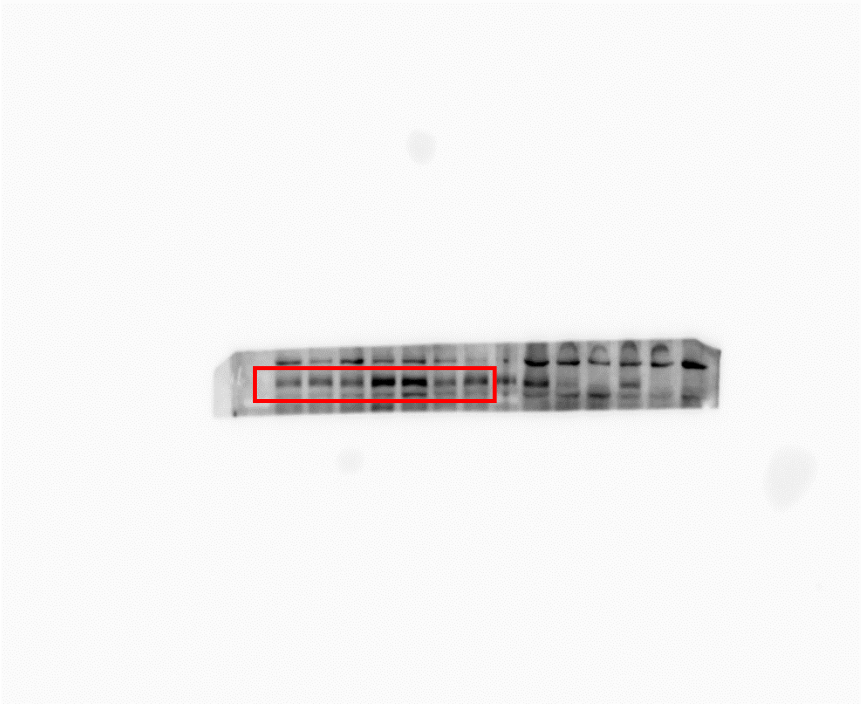

Figure 2B

Huh-7 actin

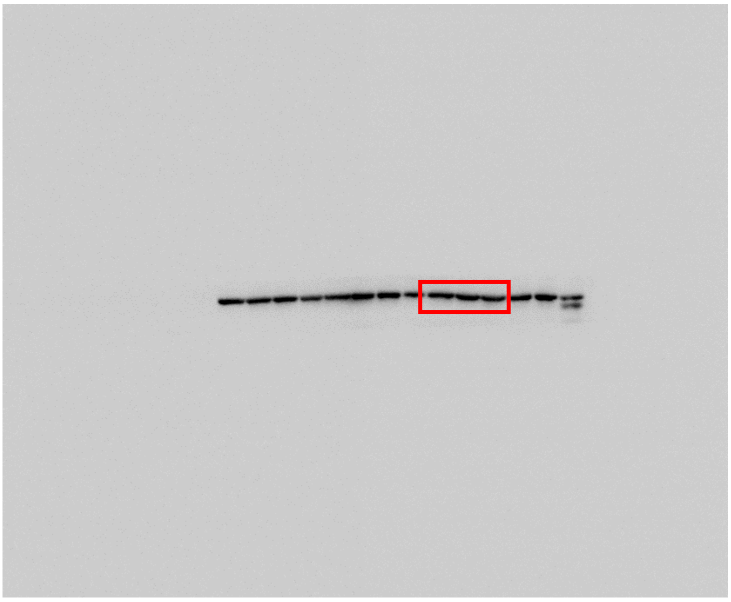

Huh-7 RNF31

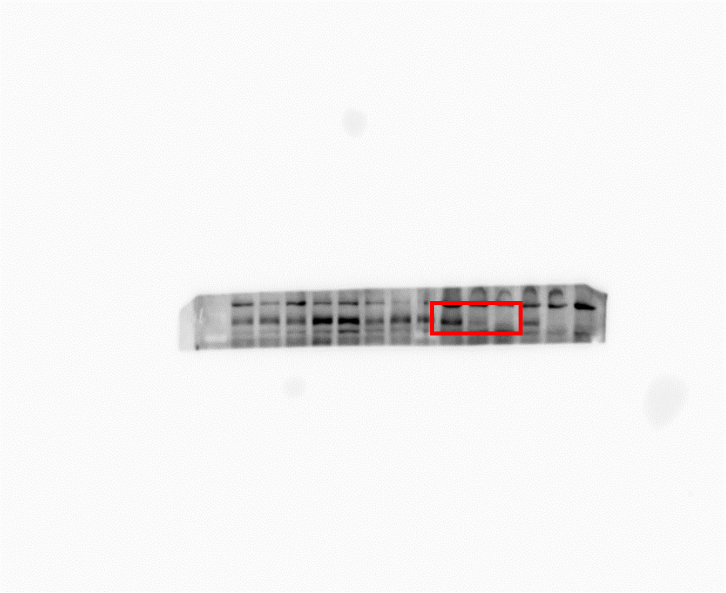

PLC/PRF/5 actin

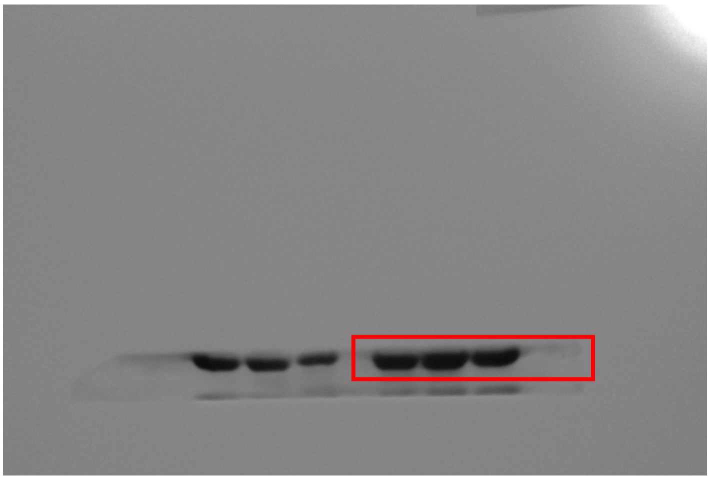

PLC RNF31

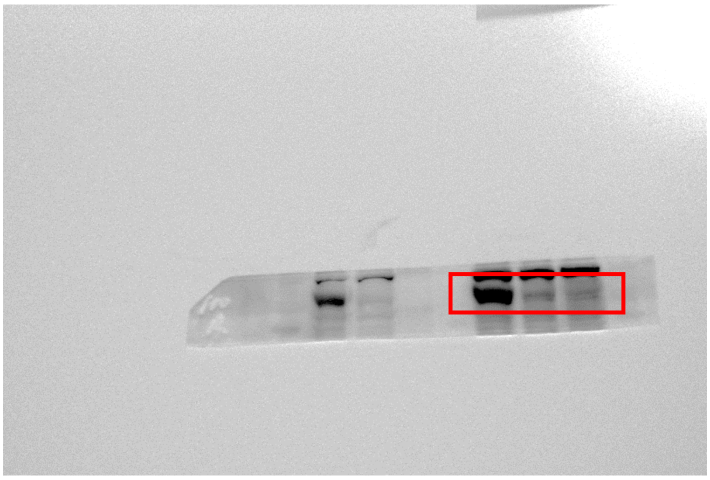

Figure 4B Huh7

RBCK1 (Left)

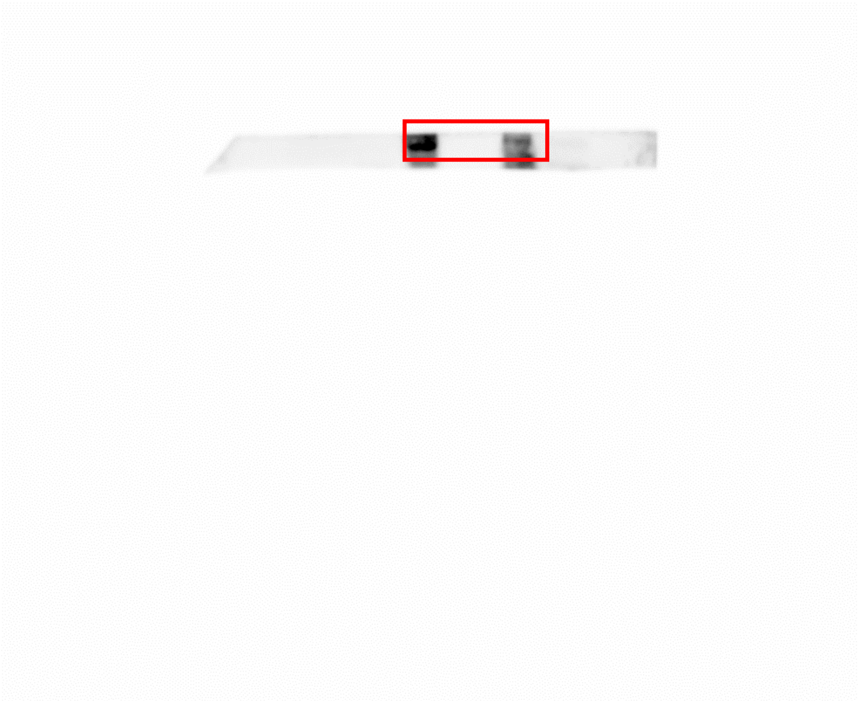

RNF31 (Left)

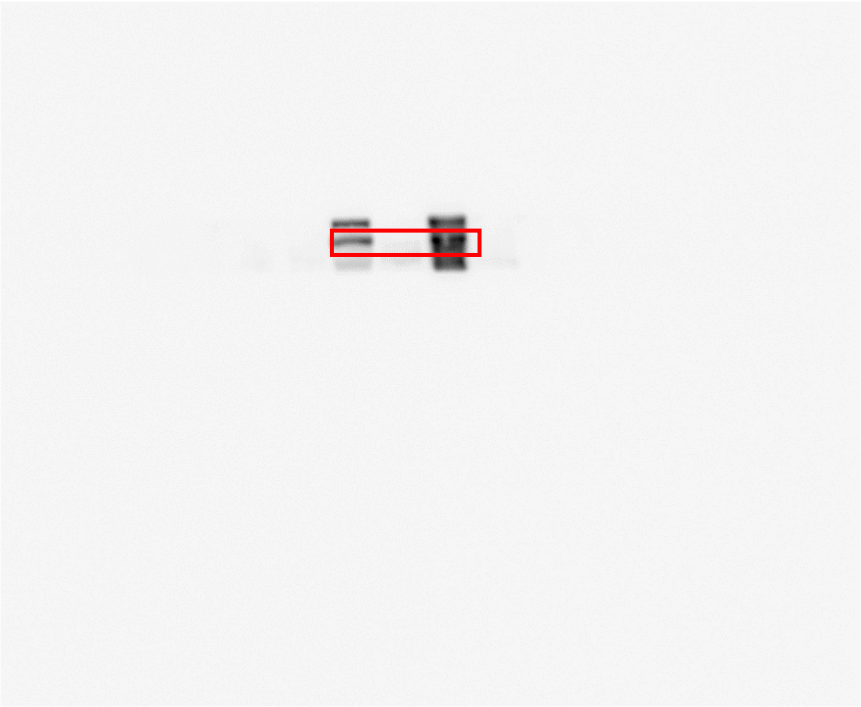

RBCK1 (Right)

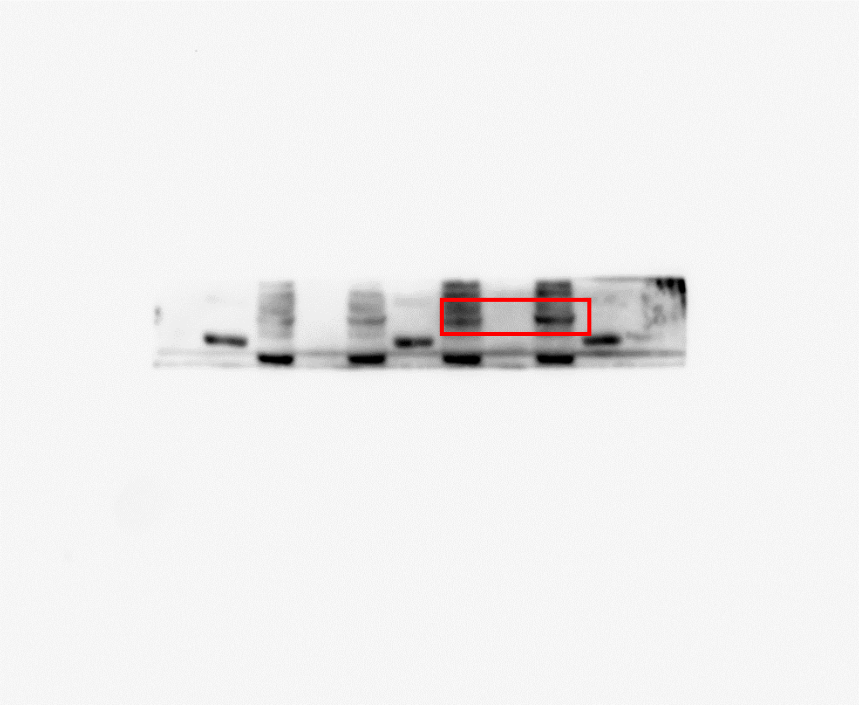

RNF31 (Right)

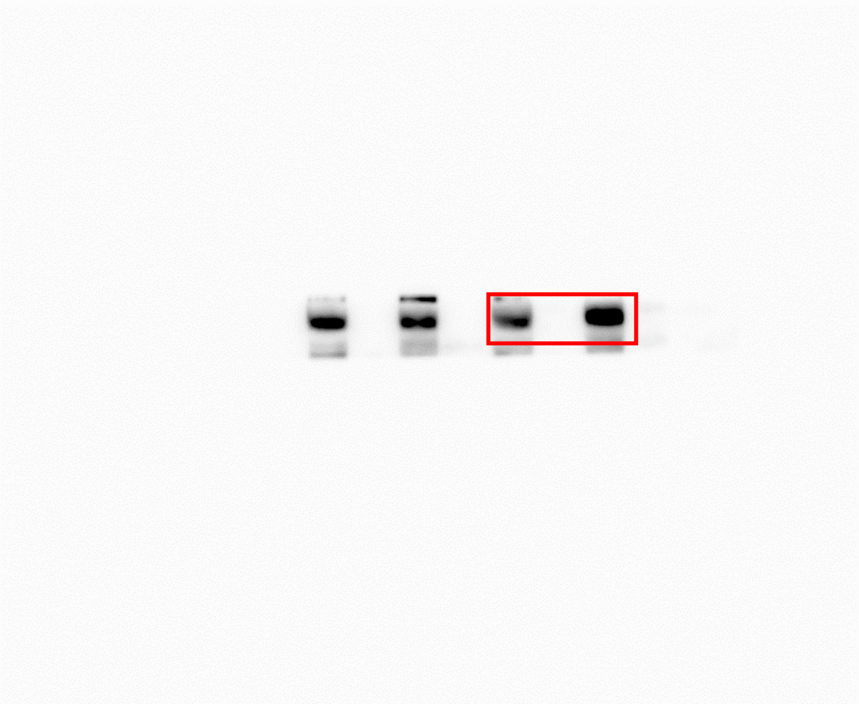

Figure 4B PLC/PRF/5

RBCK1 (Left, Right)

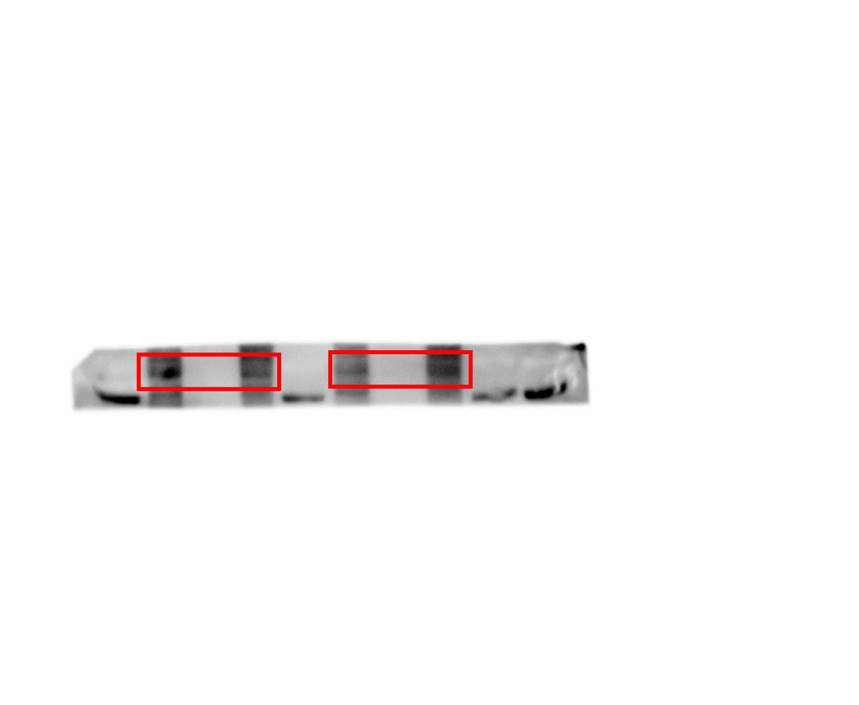

RNF31 (Left, Right)

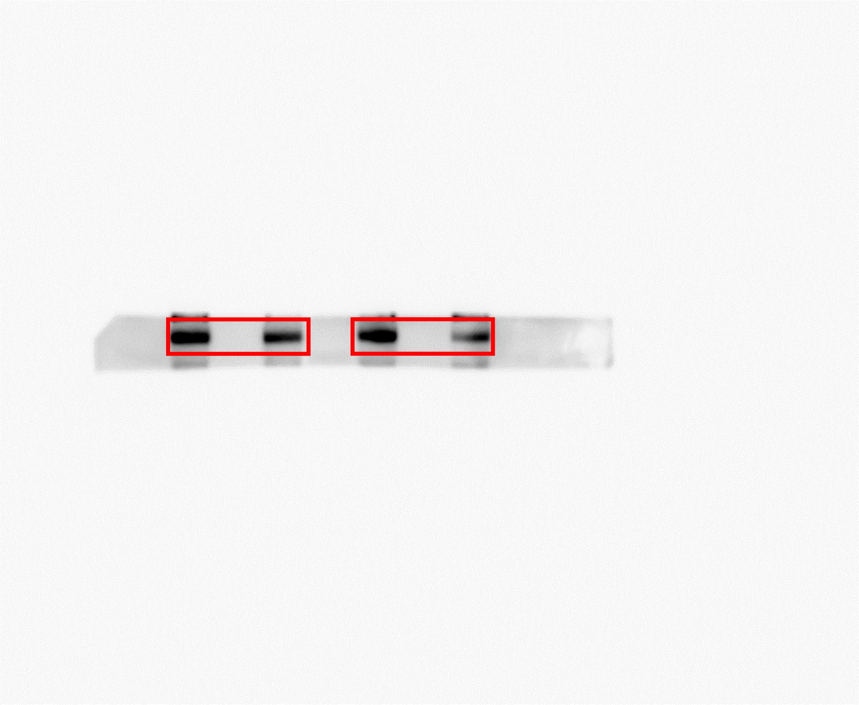

Figure 4C

HUH7

ACTIN

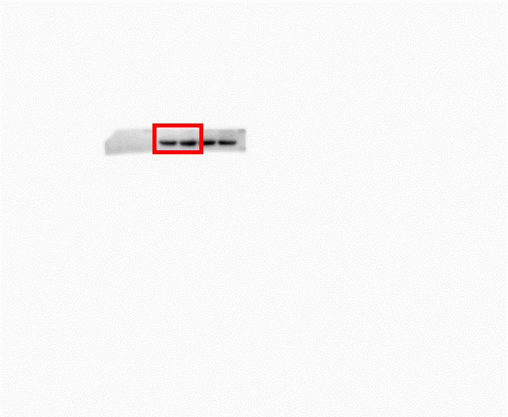

RBCK1

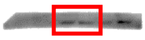

RNF31

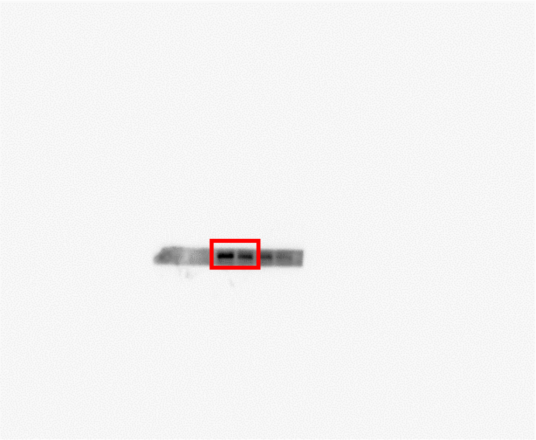

Figure 4C PLC/PRF/5

ACTIN

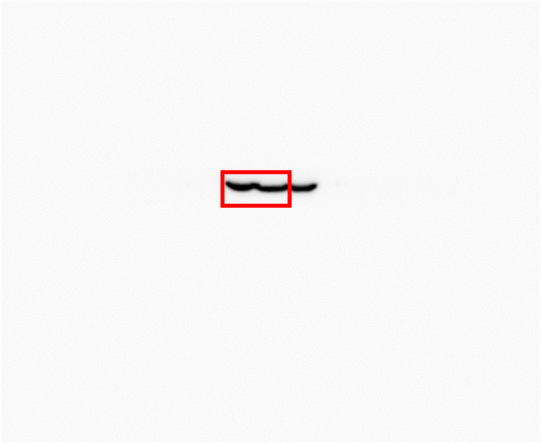

RBCK1

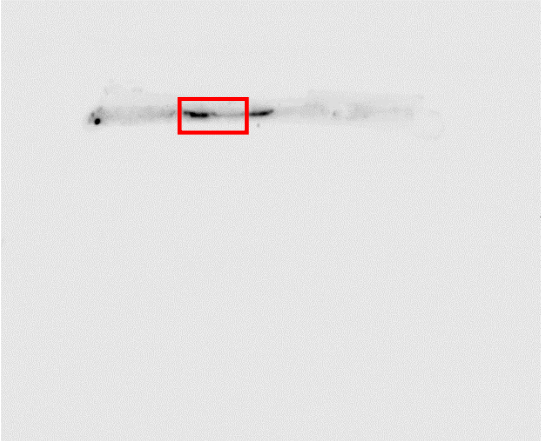

RNF31

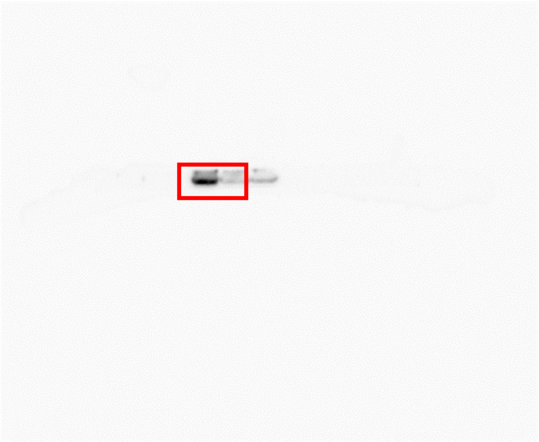

Figure 4E Huh7

ACTIN

RBCK1

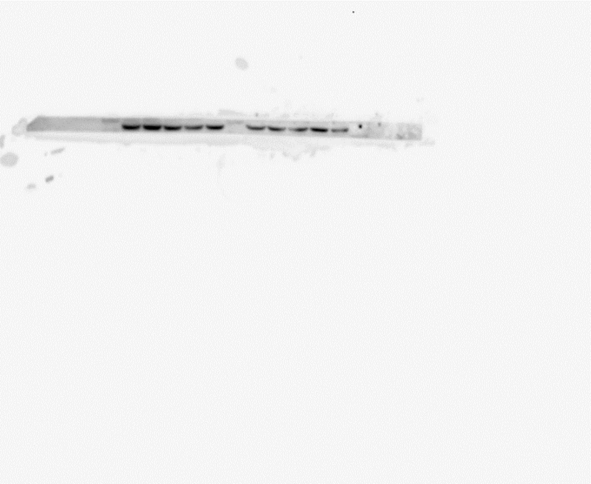

RNF31

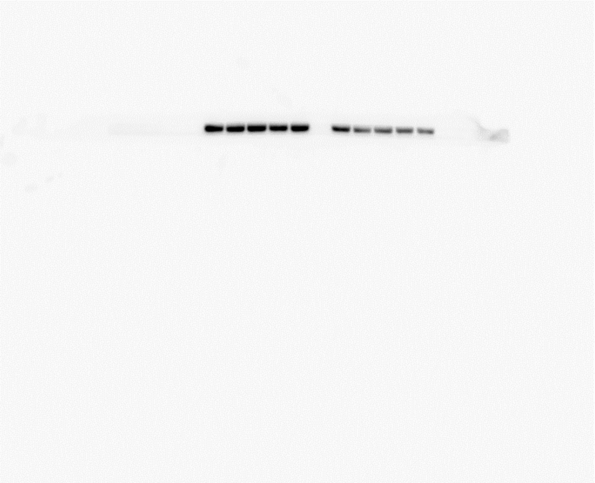

Figure 4E PLC/PRF/5

ACTIN

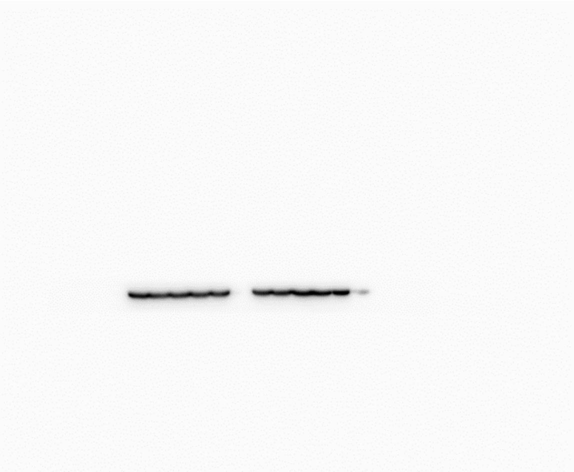

RBCK1

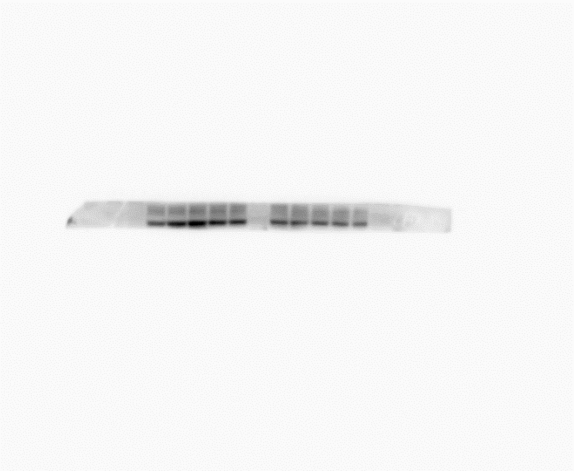

RNF31

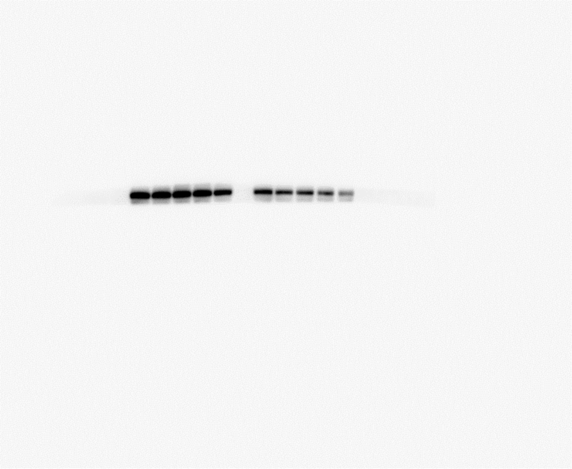

Figure 4F Huh7

ACTIN

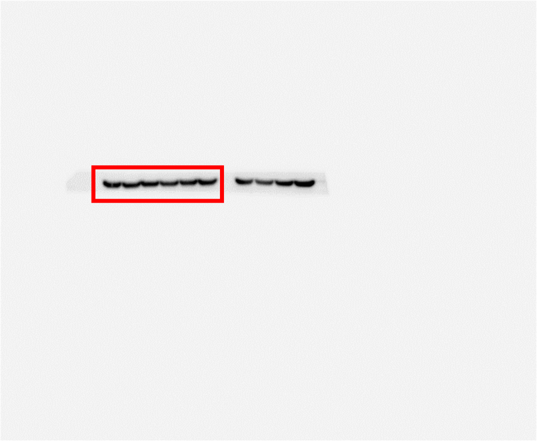

RBCK1

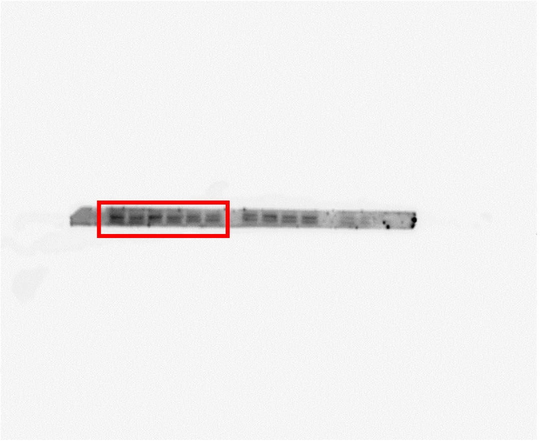

RNF31

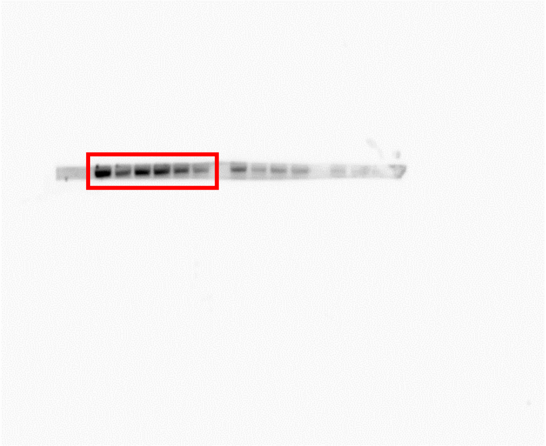

Figure 4F PLC/PRF/5

ACTIN

RBCK1

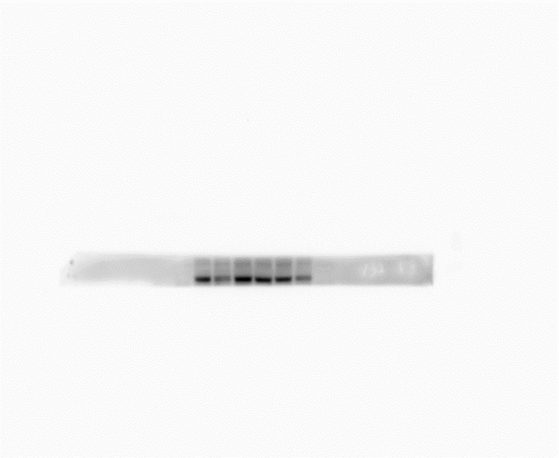

RNF31

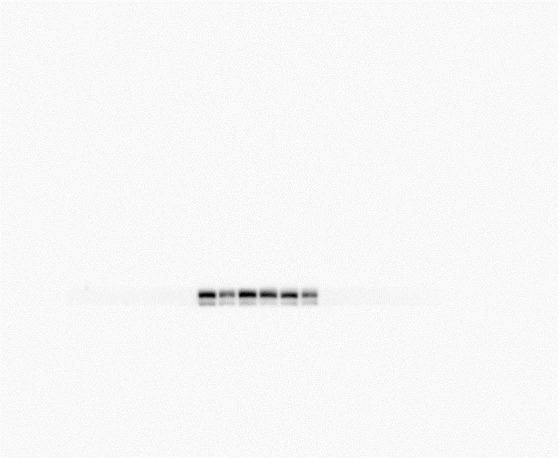

Figure 4G Huh7

Input-ACTIN

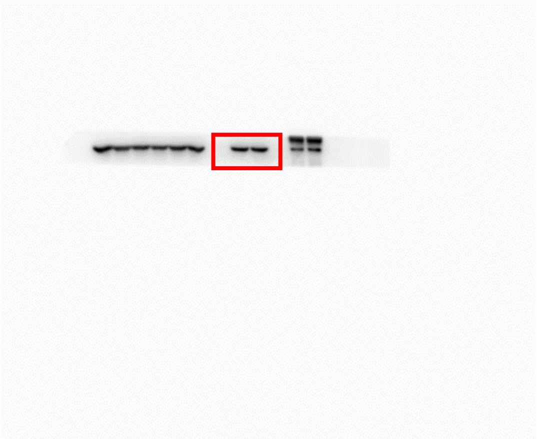

Input-RBCK1

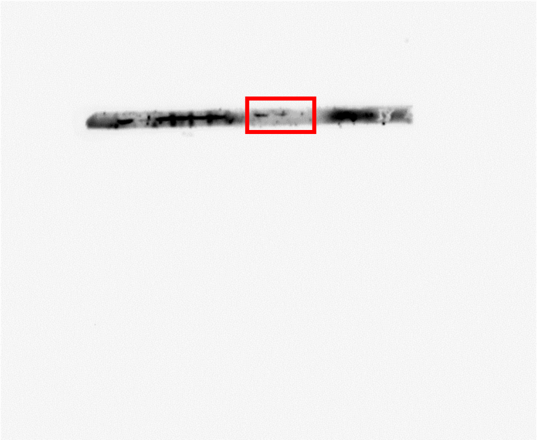

Input-RNF31

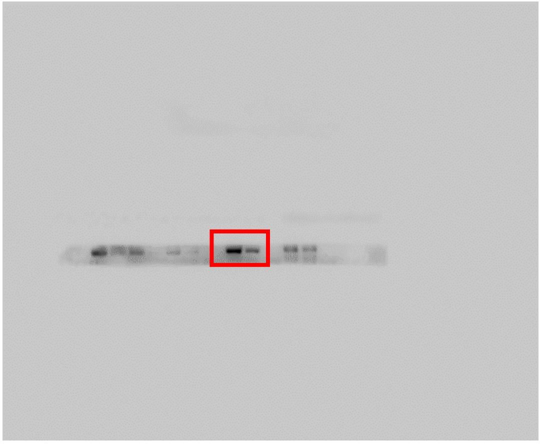

IP-RNF31

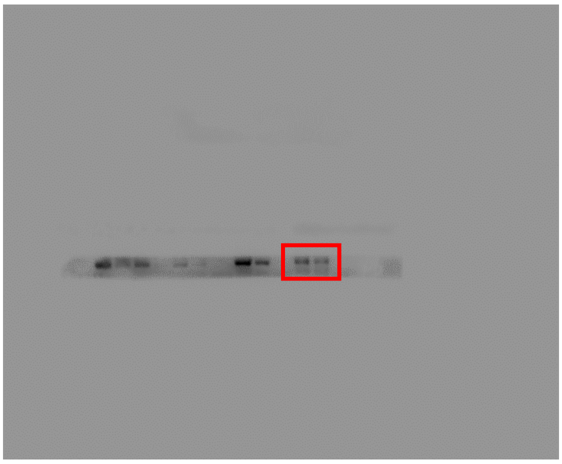

IP-Ub

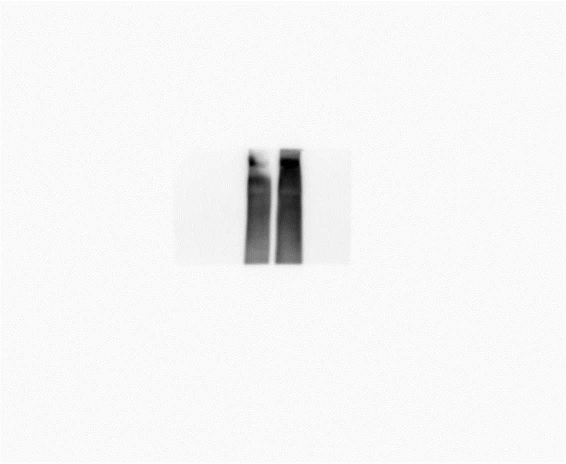

Figure 4G PLC/PRF/5

Input-ACTIN

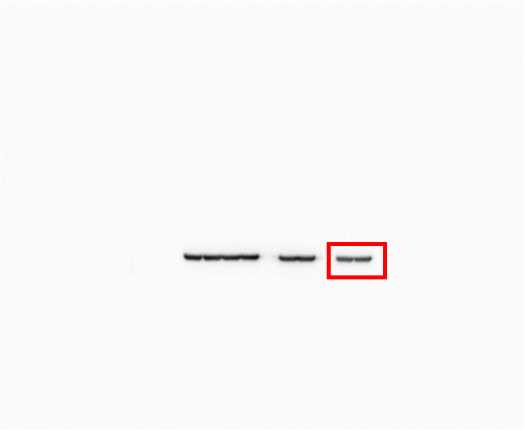

Input-RBCK1

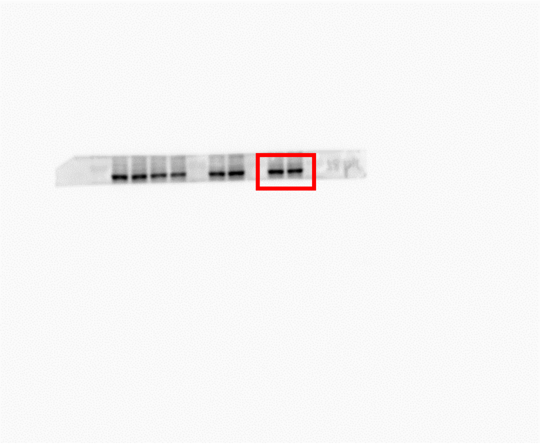

Input-RNF31

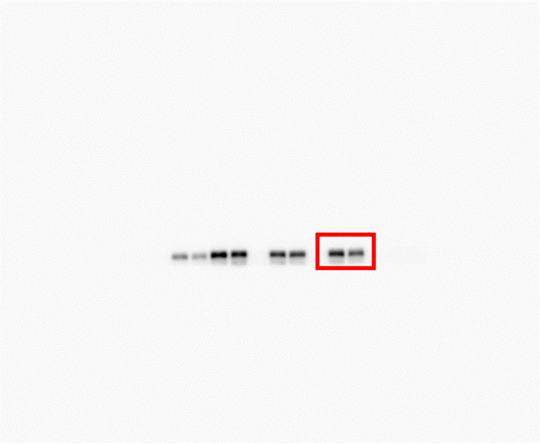

IP-RNF31

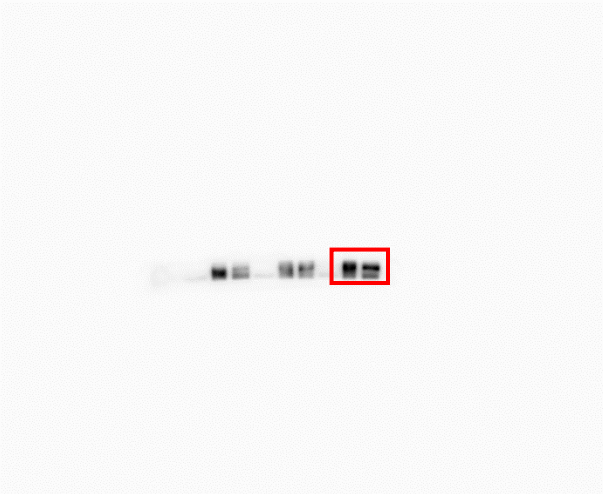

IP-UB

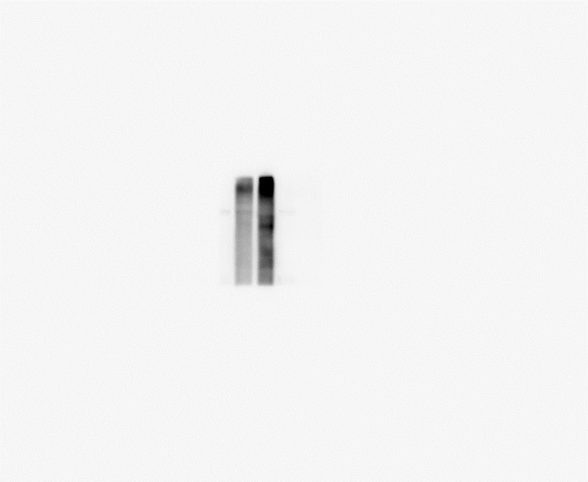

Figure 5A Huh-7

ACTIN

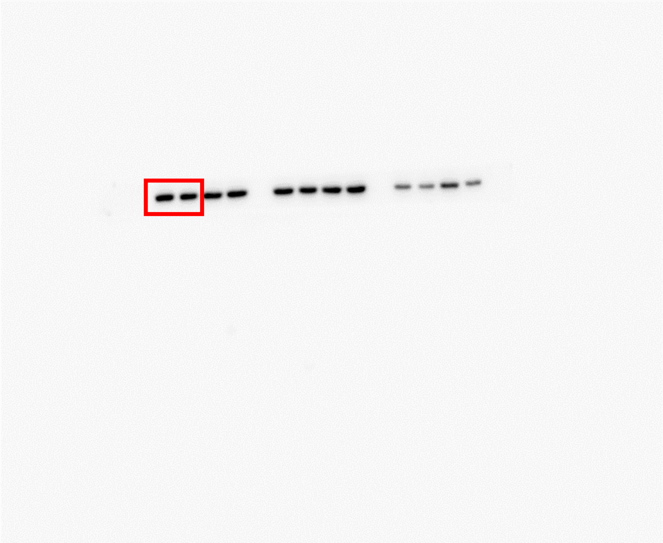

RBCK1

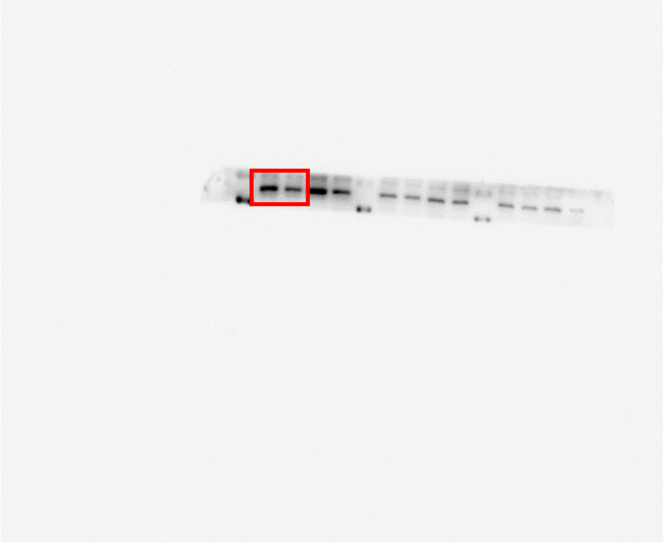

Figure 5A PLC/PRF/5

ACTIN

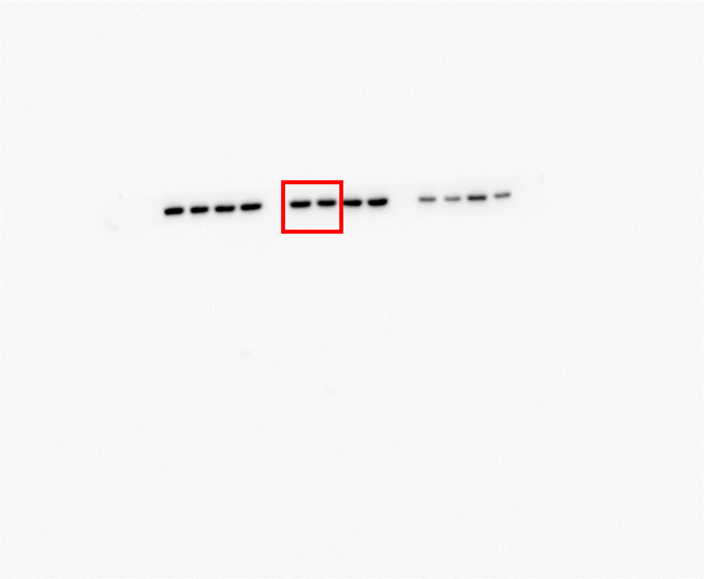

RBCK1

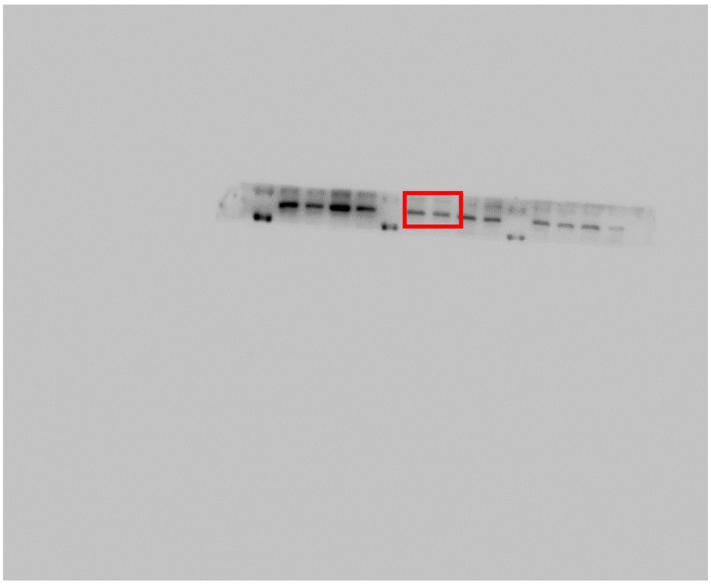

Figure 6A Huh-7

ACTIN

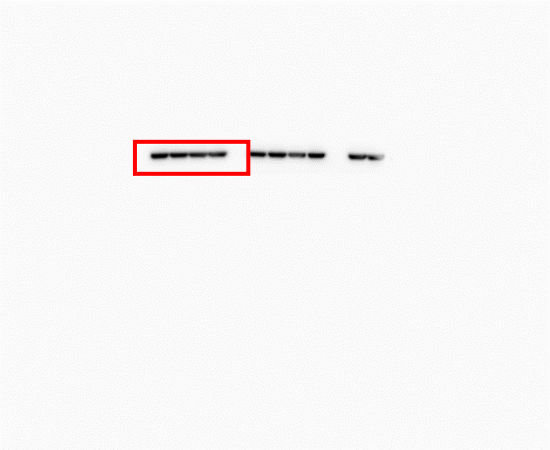

RBCK1

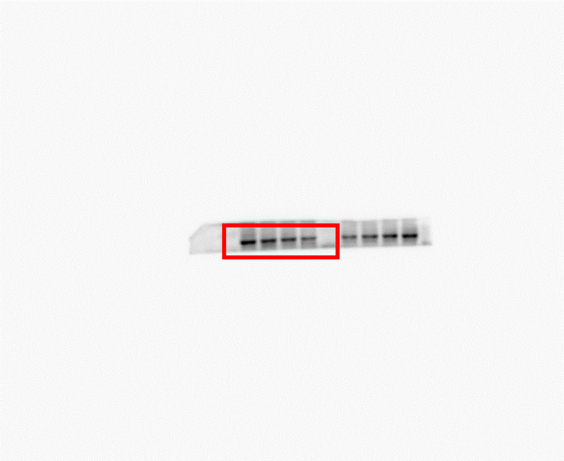

RNF31

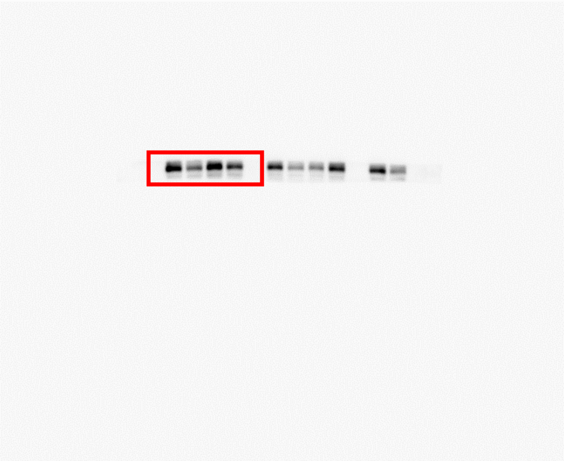

Figure 6A PLC/PRF/5

ACTIN

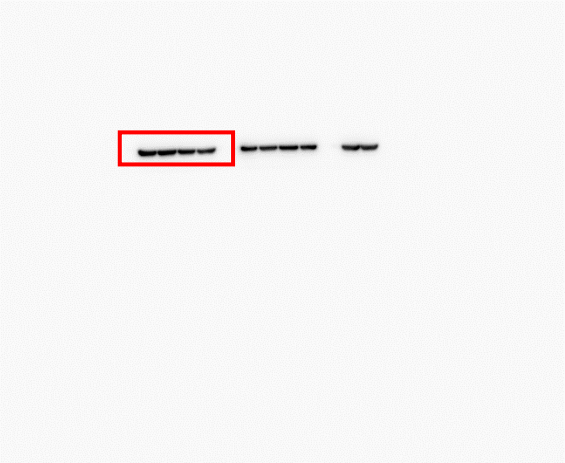

RBCK1

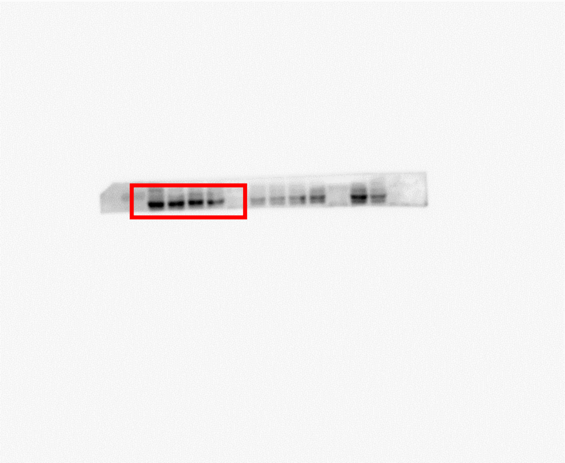

RNF31

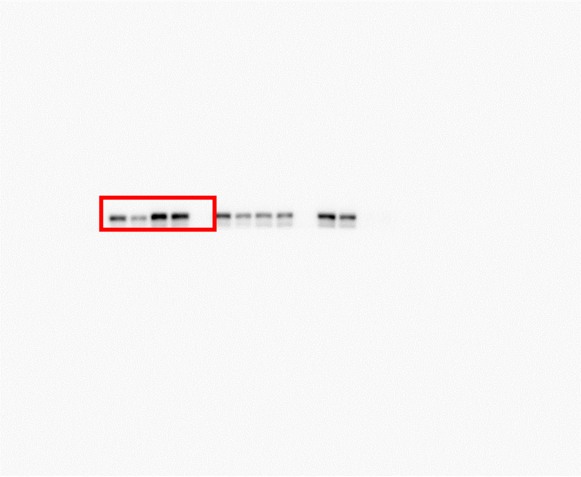

Supplementary Figure 4 Huh-7

ACTIN

RBCK1

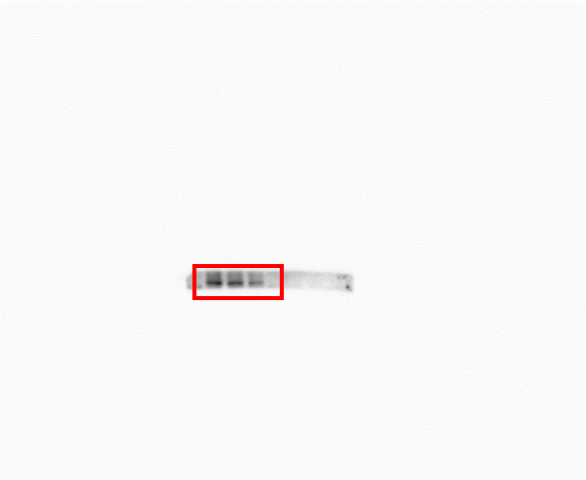

RNF31

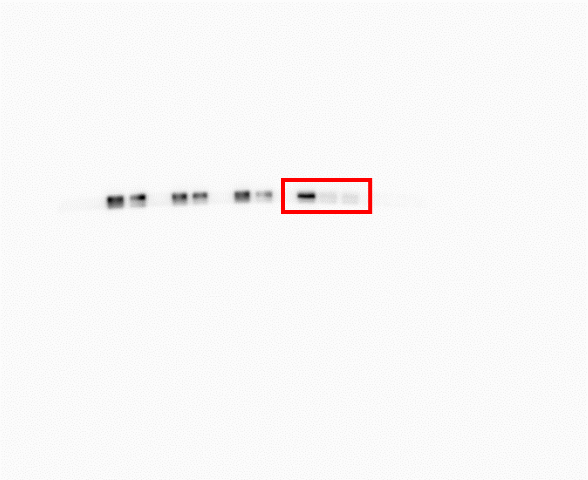

Supplementary Figure 4 PLC/PRF/5

ACTIN

RBCK1

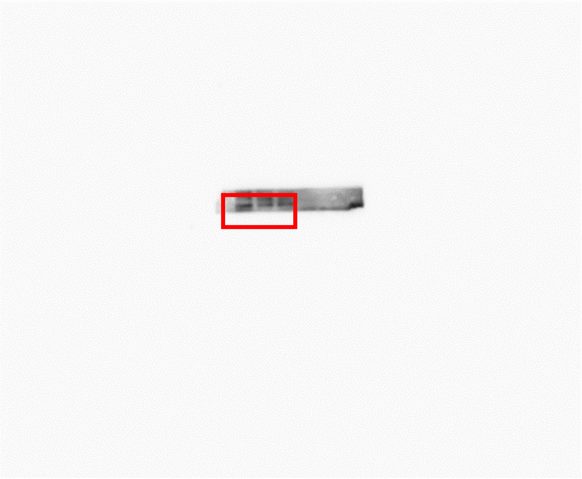

RNF31

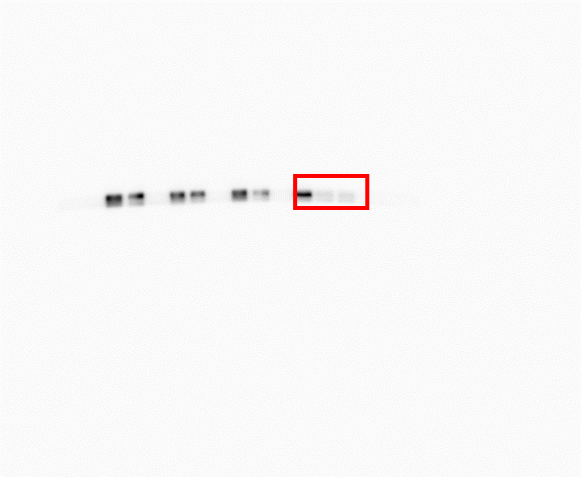

Supplementary Figure 1D P53:

ACTIN

RNF31

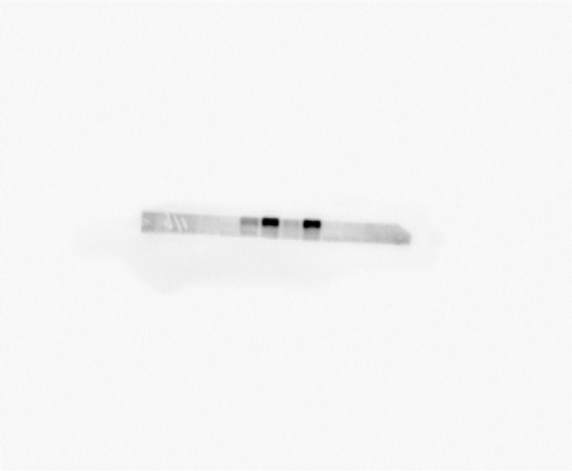

P53

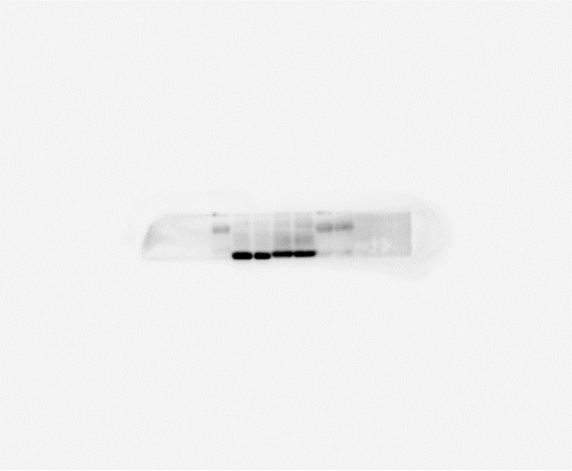

Supplementary Figure 1D PGM1, FGB, AGXT:

ACTIN

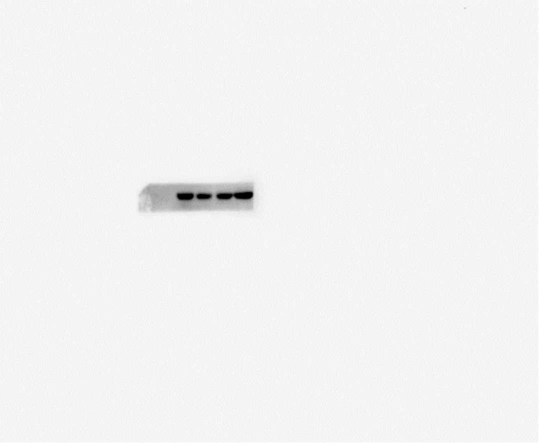

RNF31

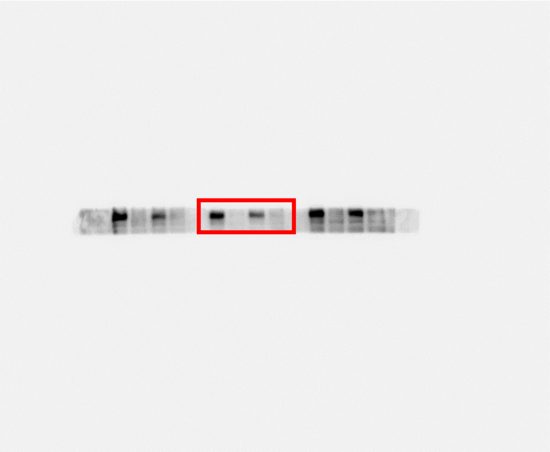

PGM1

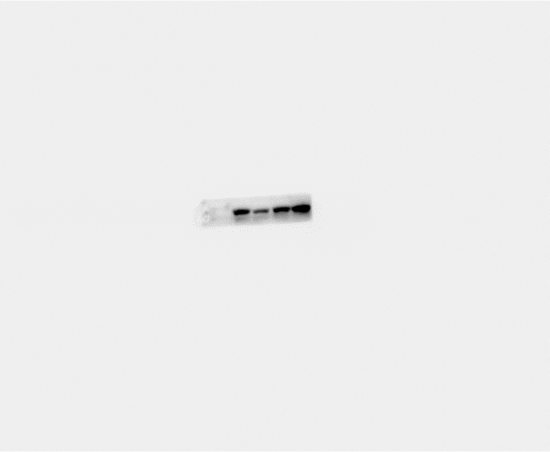

Supplementary Figure 1D PGM1, FGB, AGXT:

FGB

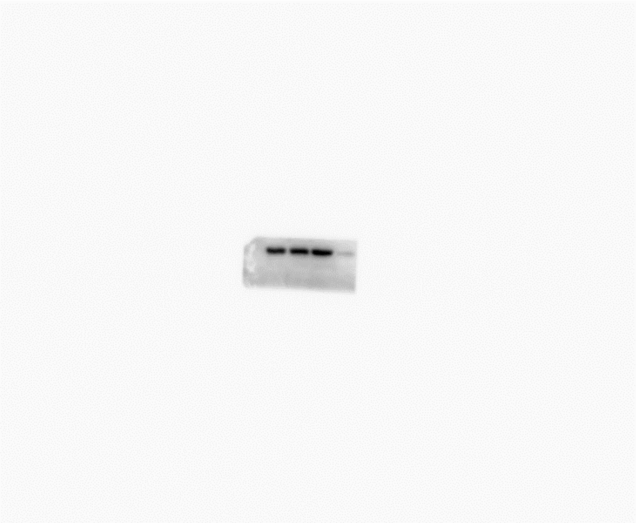

AGXT

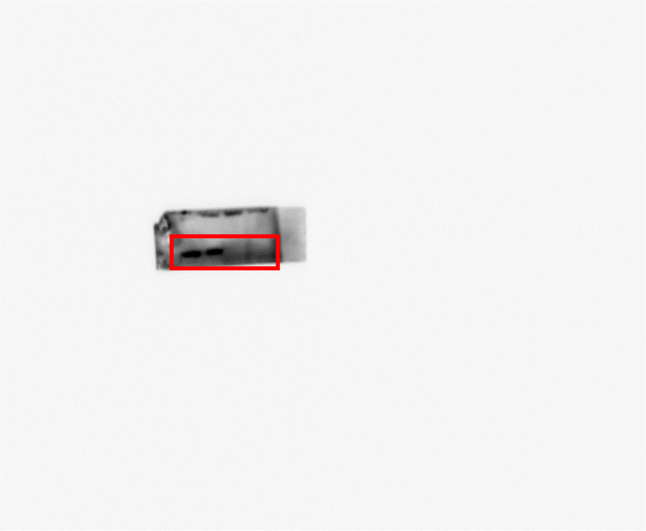

Supplementary Figure 1D ESR1, ANXA2:

ACTIN

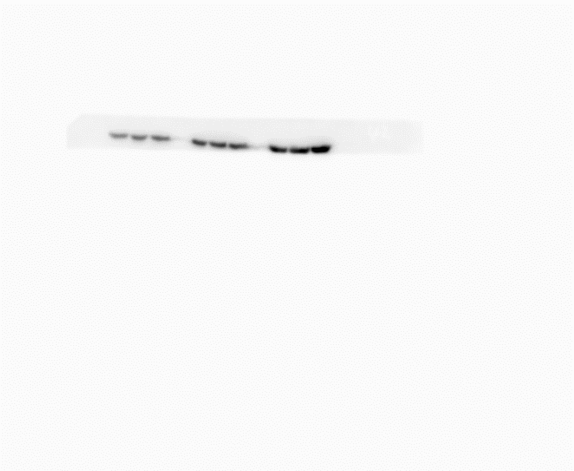

RNF31

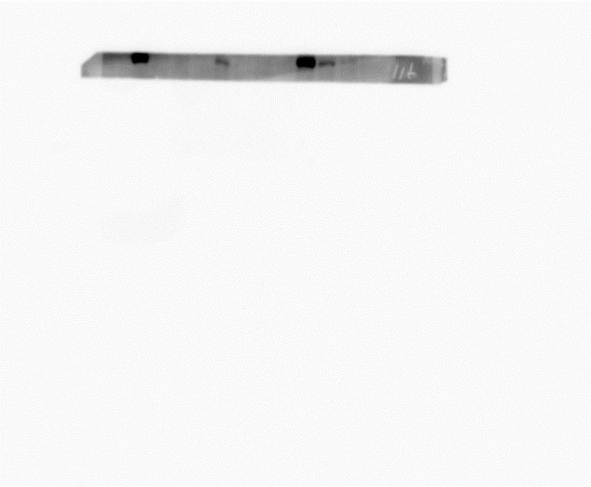

ESR1

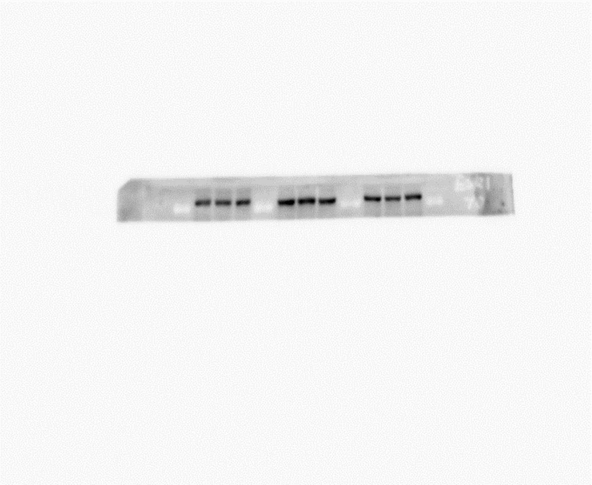

Supplementary Figure 1D ESR1, ANXA2:

ANXA2

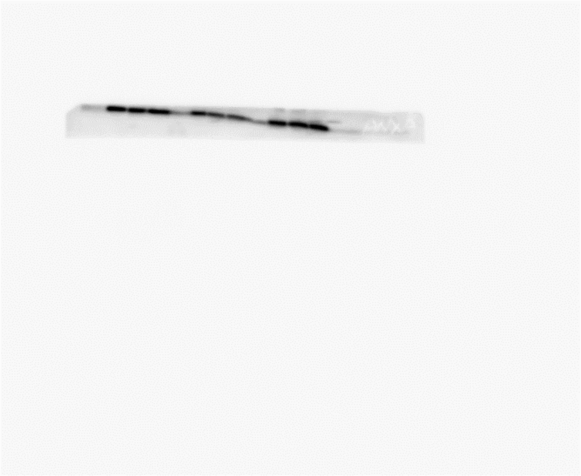

Supplementary Figure 1D EIF3F, RTN3, PRDX4：

GAPDH

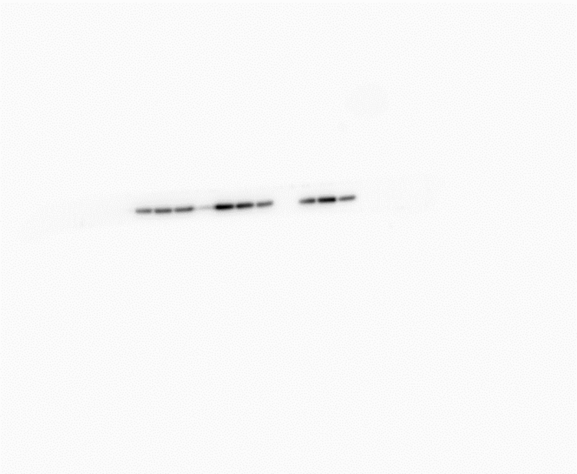

RNF31

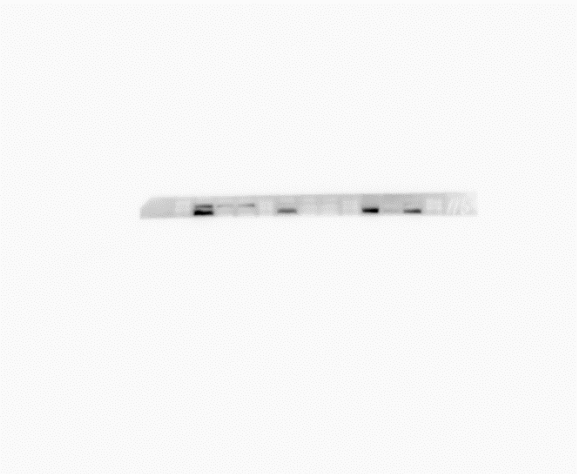

EIF3F

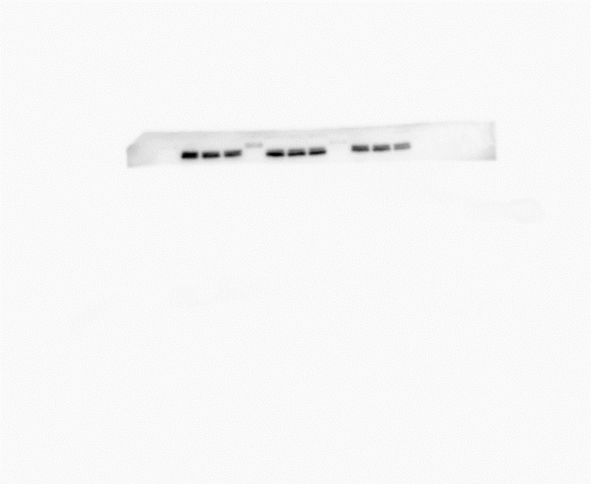

Supplementary Figure 1D EIF3F, RTN3, PRDX4：

RTN3

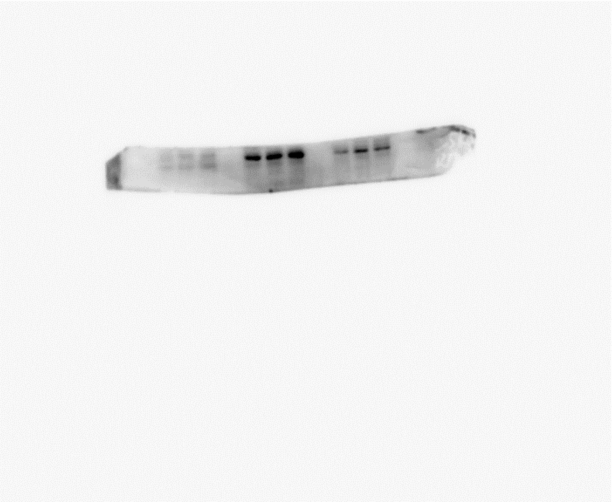

PRDX4

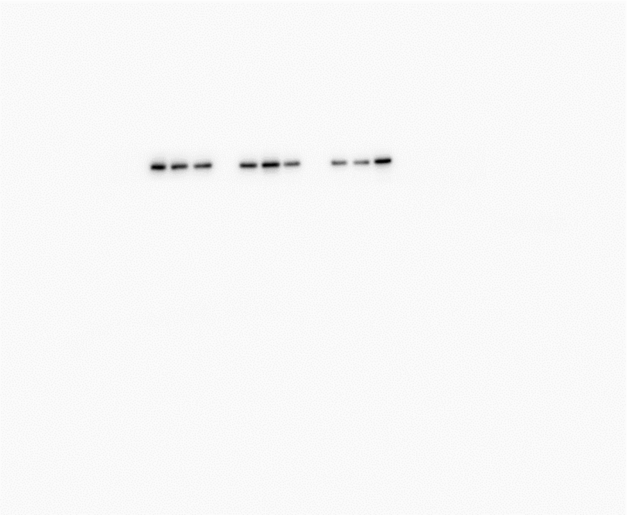

Supplementary Figure 1D HSP90AA1, SEPT2：

GAPDH

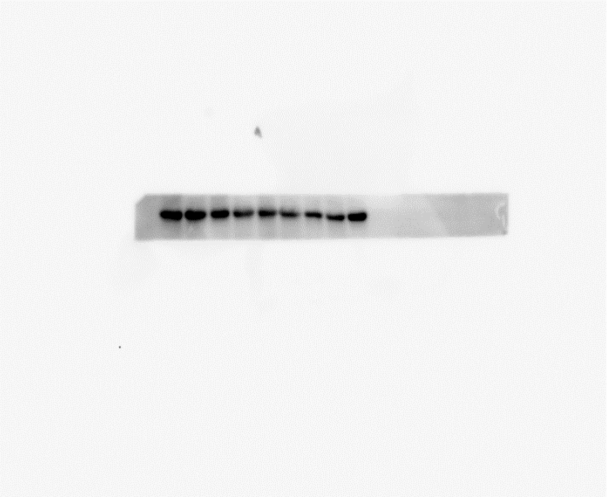

RNF31

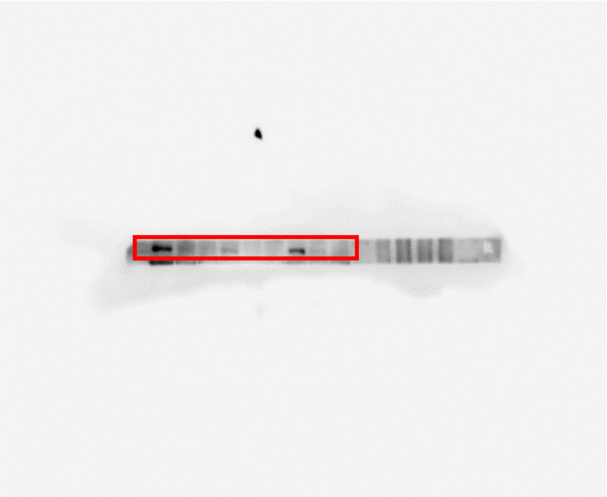

HSP90AA1

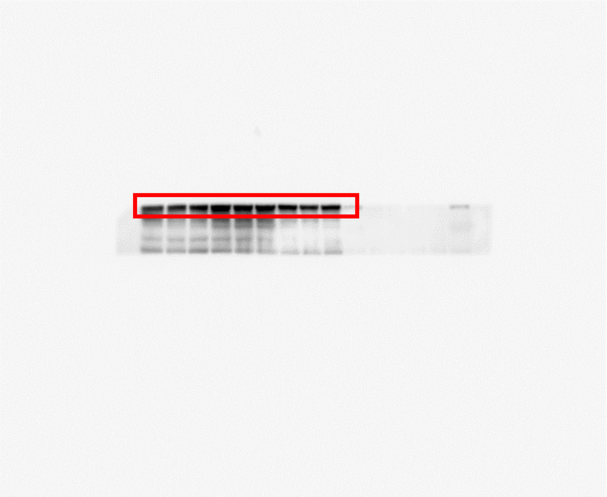

Supplementary Figure 1D HSP90AA1, SEPT2：

SEPT2

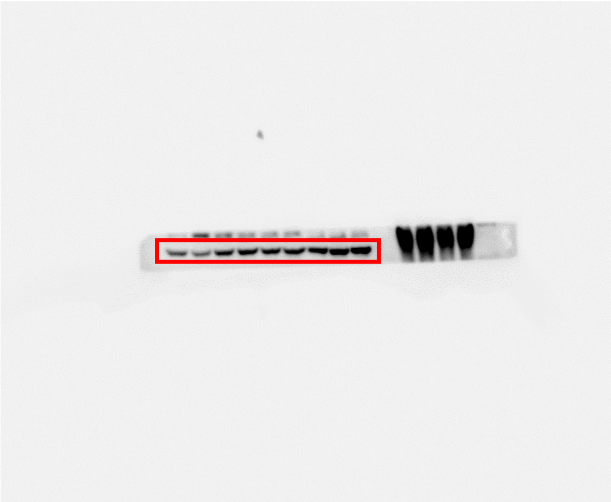

Supplement: Supplementary file 2 — Original Data File [file 41420_2022_1126_MOESM2_ESM.pdf]
